# Detecting the dominance component of heritability in isolated and outbred human populations

**DOI:** 10.1038/s41598-018-36050-7

**Published:** 2018-12-21

**Authors:** Anthony F. Herzig, Teresa Nutile, Daniela Ruggiero, Marina Ciullo, Hervé Perdry, Anne-Louise Leutenegger

**Affiliations:** 1 Inserm, U946, Genetic variation and Human diseases, Paris, France; 2 Université Paris-Diderot, Sorbonne Paris Cité, U946 Paris, France; 3 Institute of Genetics and Biophysics A. Buzzati-Traverso - CNR, Naples, Italy; 4 IRCCS Neuromed, Pozzilli, Isernia, Italy; 5 Université Paris-Saclay, University. Paris-Sud, Inserm, CESP, Villejuif, France

## Abstract

Inconsistencies between published estimates of dominance heritability between studies of human genetic isolates and human outbred populations incite investigation into whether such differences result from particular trait architectures or specific population structures. We analyse simulated datasets, characteristic of genetic isolates and of unrelated individuals, before analysing the isolate of Cilento for various commonly studied traits. We show the strengths of using genetic relationship matrices for variance decomposition over identity-by-descent based methods in a population isolate and that heritability estimates in isolates will avoid the downward biases that may occur in studies of samples of unrelated individuals; irrespective of the simulated distribution of causal variants. Yet, we also show that precise estimates of dominance in isolates are demonstrably problematic in the presence of shared environmental effects and such effects should be accounted for. Nevertheless, we demonstrate how studying isolates can help determine the existence or non-existence of dominance for complex traits, and we find strong indications of non-zero dominance for low-density lipoprotein level in Cilento. Finally, we recommend future study designs to analyse trait variance decomposition from ensemble data across multiple population isolates.

## Introduction

For a plethora of human traits, there is an observable resemblance between close relatives. This suggests the presence of genetic constituents in the architectures of such traits and leads to an obvious question: for a pair of individuals, can one describe a relationship between their degree of relatedness (genomic sharing) and the degree of similarity of their trait values? Fisher unravelled this question by proposing a decomposition of the variance of a trait, with components attributed to each individual’s genome and to the amassment of environmental exposures in each individual’s history. This genetic component of the variability is known as the heritability of the trait which Fisher connected to the correlation of trait values between relatives. Heritability has been estimated extensively for a multitude of traits and through diverse models and study designs. Importantly, the recent availability of dense genetic data in large cohorts has enabled the estimation of heritability from samples of unrelated individuals whereas previous estimations had been driven by studies of close relatives such as twins or nuclear families. A review of heritability estimation in related individuals can be found in Tenesa & Haley^1^ and a recent discussion of heritability estimation in unrelated individuals can be found in Yang *et al*.^2^.

An important distinction is to be made between broad-sense heritability (*H*^2^) and the more commonly communicated narrow-sense heritability (*h*^2^). This stems from the innovative modelling of complex traits by Fisher who demonstrated the interest of splitting the genetic variance of a trait into additive, dominant (interaction of alleles within a genotype of a single locus), and epistatic (interaction between genotypes of multiple loci) components^3^. For details on more elaborate models, we refer the reader to Abney *et al*.^4^ and Young & Durbin^5^. Briefly put, *h*^2^ describes the additive contributions of each allele received from one’s parents while *H*^2^ encompasses the effect of one’s whole genome and is the sum of *h*^2^ and the contributions of non-additive effects. For the purposes of this study, we term this non-additive fraction of variance as ‘dominant’ as we do not here consider epistasis or higher order variance terms; we will denote this component as $\,{h}_{D}^{2}$ (equal to *H*^2^ − *h*^2^). In terms of phenotypic similarities between family members, the parent/offspring correlation is equal to $\frac{1}{2}{h}^{2}$ while the sibling correlation is equal to $\frac{1}{4}{h}_{D}^{2}+\frac{1}{2}{h}^{2}$. To give clarity, we define $\,{h}_{A}^{2}=\,{h}^{2}$.

We will consider the estimation of heritability through maximum-likelihood estimation of variance parameters of linear mixed models (LMMs). For a setting of *N* individuals and *Y* a vector of observed phenotypes, we will consider the following model with fixed effects *X*and a variance-covariance structure split into genetic additive, genetic dominant, and environmental components:

1 $$Y \sim MVN({\beta }_{0}^{T}X,{\tau }_{A}K+{\tau }_{D}D+{\sigma }_{E}^{2}{I}_{N})$$

We then are able to estimate the heritabilities as follows:

2 $${H}^{2}=\frac{{\tau }_{A}+{\tau }_{D}}{{\tau }_{A}+{\tau }_{D}+{\sigma }_{E}^{2}},\,\,{h}_{A}^{2}=\frac{{\tau }_{A}}{{\tau }_{A}+{\tau }_{D}+{\sigma }_{E}^{2}},\,\,{h}_{D}^{2}=\frac{{\tau }_{D}}{{\tau }_{A}+{\tau }_{D}+{\sigma }_{E}^{2}}$$

There are various possible choices of the *N* × *N* matrices *K* and *D*. Historically, *K* and *D* are defined in terms of identity-by-descent (IBD) probabilities^4,6,7^. *K* is equal to 2*φ*, where *φ*_*i*, *j*_ is the kinship coefficient of individuals *i* and *j*, defined as the probability of two alleles, randomly sampled from each of individuals *i* and *j*, at the same locus will be IBD. *D*_*i*, *j*_ is the probability that individuals *i* and *j* share exactly two pairs of alleles IBD at a given locus. Both *φ*_*i*, *j*_ and *D*_*i*, *j*_ are themselves expressions of Jacquard’s nine coefficients of identity: ${\phi }_{i,j}={{\rm{\Delta }}}_{1}+\frac{1}{2}({{\rm{\Delta }}}_{3}+{{\rm{\Delta }}}_{5}+{{\rm{\Delta }}}_{7})+\frac{1}{4}{{\rm{\Delta }}}_{8}$, and *D*_*i*, *j*_ = Δ_1_ + Δ_7_^6^. In studies of family data or isolated populations, these coefficients have been classically estimated from pedigree information but with the advent of dense genomic information, they can now be estimated reliably from genotype data by either estimating genome-wide IBD sharing probabilities or detecting and counting IBD segments^8–10^. Such methods have also been developed for studies of unrelated individuals^11^, though the predominant approach in such studies is to use moment estimators of *K* and *D* by taking correlations between each pair of individuals’ (orthogonal) additive and dominant genetic components, respectively^12,13^. These latter estimators are known as genetic relationship matrices (GRMs) and can be used in any study design.

This leads to two distinct interpretations of the matrices *K* and *D* which both come with potential drawbacks. If IBD probabilities are used to estimate *K* and *D*, they represent the level of relatedness between pairs of individuals based on the presence of recent common ancestors but if *K* and *D* are estimated as GRMs, then they represent simply the correlation between pairs of individuals’ genotypes. For the former interpretation, coefficients of identity can only be approximated either by their expected values based on the pedigree structure linking individuals or by estimating the proportions of IBD-sharing between individuals based on their genotypes. However, exhaustive pedigree information is never available and indeed the concept of IBD is similarly problematic due to the ambiguity of how many generations to consider when looking back for evidence of shared genetic ancestors. After many generations, mutations and recombinations cause the IBD segments to become increasingly short and not completely identical and thus difficult to distinguish from background genetic variation^14–16^. For the latter interpretation involving GRMs, there is the immediate problem that such correlations are computed from a large set of variants which are not specific to the trait being studied in the hope that these variants will be representative of the unknown set of causal variants via linkage disequilibrium (LD) (correlations between variants)^17^. Consequentially, if heritability is estimated with GRMs, it corresponds to only a proportion of the phenotypic variation coming from the subset of causal variants that are in LD with the genotyped variants^18^. This can lead to downwardly biased estimate of heritability as causal variants may often be held at low frequencies by selection^19,20^ and so will be in weak LD with common genotyped variants. Furthermore, if there exist relatively few causal variants, the large numbers of non-causal variants used to estimate the genetic correlations might mask the desired correlation of causal variants between individuals^21^. Genomic-based IBD methods applied to unrelated individuals has been suggested as an approach to improve upon genetic correlation methods as detected stretches of IBD can cover some un-typed genetic variation^11^.

The main motivation for employing GRMs is that this allows for the estimation of heritability from unrelated individuals, thus leveraging data from large cohorts and avoiding shared environment biases^13,22^. However, there has been a trend towards using genomic-based estimates even when pedigree data is available due to the increased precision of relatedness estimation from genetic data, both in human studies^16,23–27^ and in animal/plant studies^28–31^.

For complex human traits, it has been suggested that one can assume that any contributions from non-additive genetic components $({h}_{D}^{2})$ are relatively small compared to the additive genetic components^32^ and thus often only estimates of ${h}_{A}^{2}$ are presented. In a recent study, Zhu *et al*.^12^ illustrated this characterization of diminutive dominant genetic variance for 79 traits in two large samples of unrelated individuals. This result was then re-enforced in Nolte *et al*.^33^. Yet, many others have presented incongruent results on this subject. Chen *et al*.^34^ compared the same approach as Zhu *et al*.^12^ with a twin-based analysis and concluded that whilst the genetic variances of 19 traits were predominantly additive, dominant genetic components were nonetheless more prominently apparent than when described elsewhere. Aside from these studies, dominance heritability estimation using GRMs has rarely been carried out, and the authors who are more interested in dominance tend to rely on family data^35,36^. Of particular note is the observation that significant non-additive genetic components for many traits have been found in some studies on population isolates: Abney *et al*.^37^, Pilia *et al*.^38^, and Traglia *et al*.^39^ (Table 1).

**Table 1:** Published results for additive and dominant genetic variability from various study designs.

Phenotype	Abney, McPeek, & Ober^37^, *N* = 806, Isolate (1)		Pilia *et al*.^38^, *N* = 6,148, Isolate (1) (2)		Traglia *et al*.^39^, *N* = 1,803, Isolate (1) (2)		Zaitlen *et al*.^41^, *N* ≈ 15,000, Extended Genealogies (3)		van Dongen *et al*.^35^, *N* ≈ 7,500, Twin Study (4)		Chen *et al*.^34^, *N* = 7,740, Twin Study (5)		Chen *et al*.^34^, *N* = 5,779, Outbred (5) (6)		Zhu *et al*.^12^, *N* = 8,682, Outbred (6)		Nolte *et al*.^33^, *N* = 13,436, Outbred (6)	
	${h}_{A}^{2}$	${h}_{D}^{2}$	${h}_{A}^{2}$	${h}_{D}^{2}$	${h}_{A}^{2}$	${h}_{D}^{2}$	${h}_{A}^{2}$	${h}_{D}^{2}$	${h}_{A}^{2}$	${h}_{D}^{2}$	${h}_{A}^{2}$	${h}_{D}^{2}$	${h}_{A}^{2}$	${h}_{D}^{2}$	${h}_{A}^{2}$	${h}_{D}^{2}$	${h}_{A}^{2}$	${h}_{D}^{2}$
Height	—	—	0.77	0.23 *	0.78	0.22 *	—	—	0.81	0.09	0.77	0.09*	0.62	0.00	0.48	0.02	0.49	0.00
BMI	0.54	0.00	0.36	0.32 *	0.33	0.17	0.16	0.09	0.41	0.37	0.28	0.41*	0.21	0.02	0.23	0.15*	0.25	0.02
TGLY	0.37	0.00	0.30	0.42 *	0.39	0.35 *	—	—	0.33	0.25	0.42	0.14	0.31	0.28*	—	—	0.19	0.01
HDL	0.63	0.00	0.47	0.11	0.62	0.00	0.42	0.14*	0.40	0.27	0.66	0.00	0.24	0.01	0.25	0.07	0.19	0.00
Total Chol	—	—	0.38	0.29 *	0.23	0.77 *	—	—	0.51	0.16	0.28	0.19*	0.15	0.00	0.21	0.01	0.23	0.00
LDL	0.36	0.60 *	0.37	0.27 *	0.33	0.66 *	0.20	0.26*	0.51	0.18	0.23	0.24*	0.16	0.00	0.26	0.02	0.27	0.00

*Estimates of ${h}_{D}^{2}$ presented as statistically significant at the 5% level.

‘—’ Trait not studied for dominance in the article.

(1) Estimates based on estimating *K* and *D* from expected proportions of identity-by-descent (IBD) sharing coming from pedigree information.

(2) The depth of pedigree information in these studies did not allow the differentiation between a dominance model (including non-additive genetic variation) and a household model (including an effect of shared environment between siblings).

(3) The authors of this study analysed a large sample from the Icelandic population for whom extensive pedigree data was available, Matrices *K* and *D* were estimated by locating and counting stretches of IBD between pairs of individuals.

(4) This study analyses a large cohort of monozygotic and dizygotic adult twins. Standard errors are only presented for broad-sense heritability, though it is likely that the estimates for ${h}_{D}^{2}$ for all traits other than height were significantly different to zero.

(5) The authors of this study performed separate analysis, firstly a twin based study using structural equation methods with adjustments for reported levels of time spent in a shared environment between twins, and secondly a study of a large sample of unrelated which included one individual out of most twin pairs in the first analysis.

(6) Estimates based on calculating correlations between additively and non-additively coded genotypes to compute matrices *K* and *D*.

Abbreviations: BMI: Body-mass index; TGLY: Triglycerides; HDL: High-density lipoproteins; Total Chol: Total cholesterol; LDL: Low-density lipoproteins; *N*: Sample size.

An isolate is characterized as a population arising from a small group of founders and experiencing subsequent demographic growth in isolation. Such populations will include pairs of distantly related individuals who nonetheless share long haplotypes IBD, and may even share both haplotypes IBD in some regions. The presence of both pairs of closely related individuals and pairs of cryptically related individuals suggests that isolates could be ideally suited to heritability analyses. Furthermore, isolates are of interest for assessing the existence of genetic components as one can assume that less heterogeneity in environmental exposures will be present in the population.

Studying dominance in samples of human twins or siblings can be problematic due to confounding between the sharing of genotypes and shared environmental factors^1,34^. In a large population isolate, such confounding had been deemed as unlikely to arise due to the extensive range of possible degrees of relatedness between individuals^37,40^. However, the presence of numerous sibling pairs in the sample could easily lead to confounding with the proportions of sharing two alleles IBD (IBD = 2) and indeed such confounding between estimates for dominance and shared environmental factors between relatives has recently been observed by Zaitlen *et al*.^41^ who performed a study on extended genealogies from the Icelandic populations, itself a moderate isolate.

Genetic dominance has often been considered in the study of various animal species (mammals, poultry, and fish are most commonly studied). Here, by design, confounding with shared environmental factors can often be avoided and extensive and highly accurate pedigree data can be recorded. For many traits, dominance heritability is often found to be significantly different from zero and the inclusion of dominance has been shown to give improved performance of prediction models in animal studies^42–47^. Negative results regarding the improvement of prediction given by including genetic dominance have also been presented (eg. Heidaraitabar *et al*.^48^) and indeed debate continues in regards to the practical value of non-additive variation; for recent reviews we refer the reader to Varona *et al*.^49^ and Wolak & Keller^50^. The increased interest in non-additive variation in this domain suggests that there may be value in not discounting such variation in human studies.

We propose to compare heritability estimations in a range of simulated study designs in order to contrast studies in population isolates and in samples of unrelated individuals. In this way we hope to determine whether the differences between studies in isolates and in unrelated samples stem from particular trait architectures, specific population characteristics, or non-equivalence between interpretations of heritability in differing study settings. We will also assess different methods for estimating the matrices *K* and *D* in an isolate as well as the effect of shared environmental factors between siblings on the estimation of ${h}_{D}^{2}$ in an isolate. We then proceed to analyse anew the six complex traits displayed in Table 1 in the genetic isolate of Cliento in Southern Italy where we will validate conclusions from our simulation study and search for evidence of significant non-additive genetic components.

## Results

### Effect of population structure

**Figure 1:**
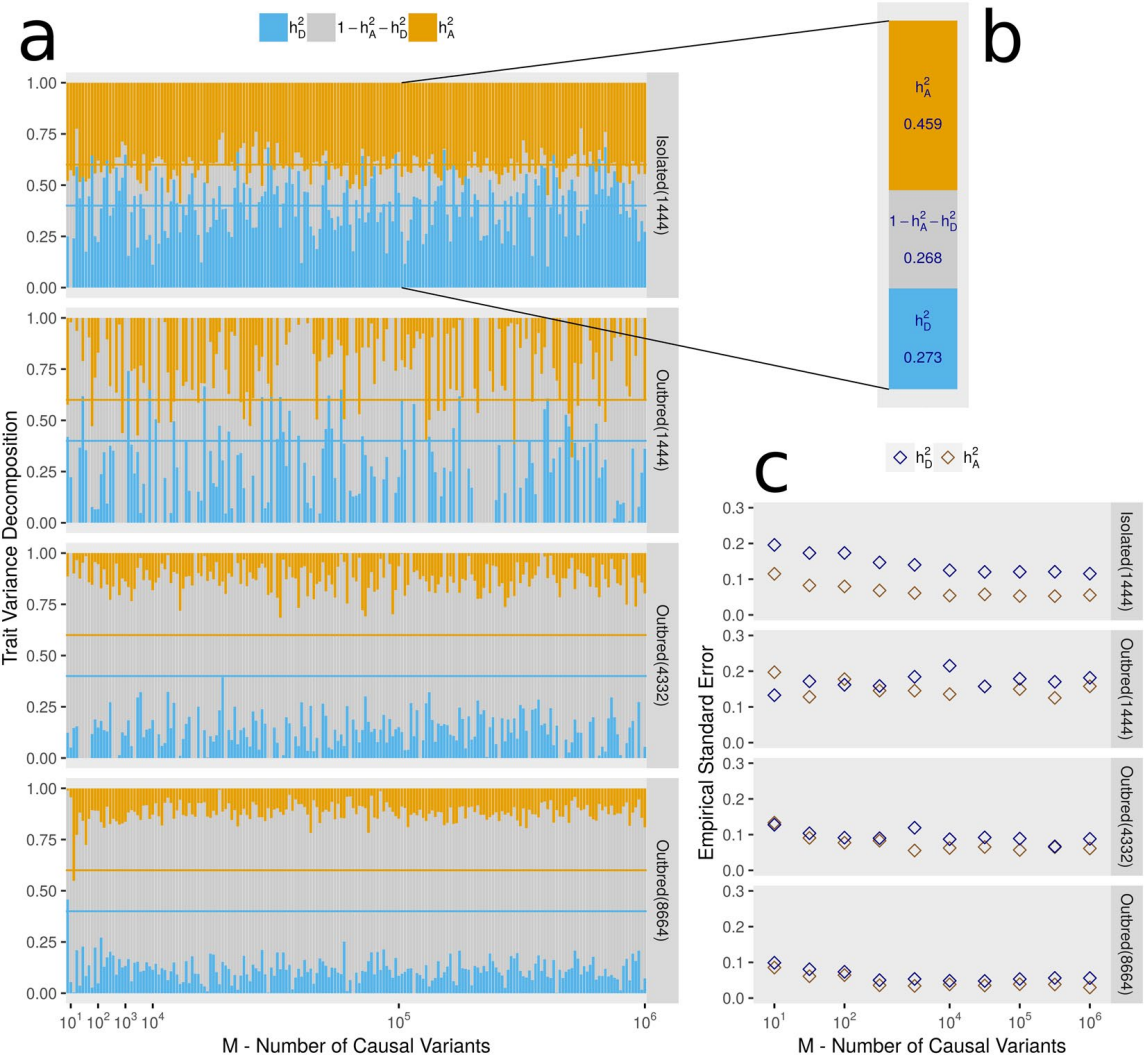
Estimating heritability components in simulated populations with different structures. (**a**) Maximum Likelihood Estimates (MLEs) of ${h}_{A}^{2}\,(gold)$ and $\,{h}_{D}^{2}\,(blue)$ are presented for each simulated phenotype by vertical descending gold and ascending blue bars respectively. The middle grey bars represent the remaining environmental variation $(1-{h}_{A}^{2}-{h}_{D}^{2}).$ Each phenotype was simulated using different numbers of causal variants (*M*) for each variance component which corresponds to the x-axis. Causal variants are mostly rare, as they are selected completely at random (Causal Variant Scenario A). All MLEs are displayed for the 4 populations either Isolated(*N*) or Outbred(*N*), where the value of *N* denotes the sample size. Horizontal gold and blue lines indicating the values used for simulation $({h}_{A}^{2}=0.4,\,{h}_{D}^{2}=0.4)$. Matrices *K* and *D* were calculated using roughly 5.8 million frequent UK10K positions. A missing bar for ${h}_{A}^{2}$ or $\,{h}_{D}^{2}$ indicates the maximum likelihood estimate of the parameter was zero. (**b**) An example of one set of MLEs from section A is given for the population Isolated(1444) and a value of *M* of 10^5^. (**c**) Gold and blue diamonds represent the empirical standard errors of the MLEs for a selection of values of *M*. Simulation repeated 500 times.

**Figure 2:**
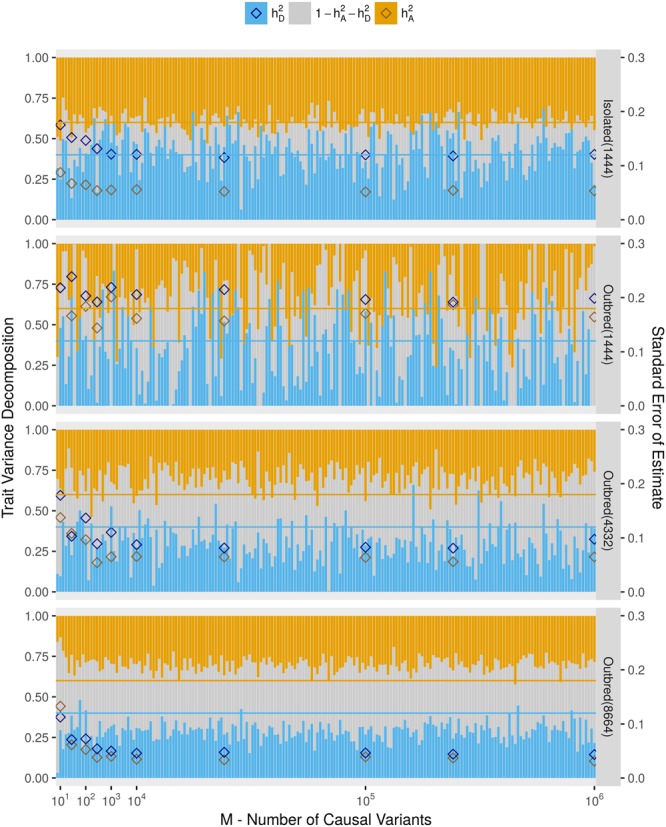
Heritability estimates when causal variants are non-rare. Here, phenotypes are simulated by choosing causal variants that are all non-rare, as they are selected to have MAF > 0.01 (Causal Variant Scenario B). Legends and the configuration of this plot are identical to those of Fig. 1A. Here, and for subsequent figures, we overlay the empirical standard error estimates, whose values correspond to the second y-axis on the right of the figure.

We assessed the ability of an LMM to detect the additive and dominant genetic variance components in four simulated populations, including firstly one population labelled “Isolated(1444)” which mimics the population structure of the genetic isolate of Cilento from Southern Italy (this cohort is described fully in the Methods section), along with three simulated outbred populations, “Oubred(1444)”, “Outbred(4332)”, and “Outbred(8644)” where the numbers in parentheses indicate the sample sizes. All populations are formed from mosaic haplotypes arising from the UK10K imputation panel^51^. We simulated phenotypes with the following characteristics: ${h}_{A}^{2}={h}_{D}^{2}=0.4$, *M* causal additive variants, and *M* causal dominant variants. Causal variants are selected at random and effect sizes are drawn from normal distributions. Full details of the simulation of genotypes, phenotypes, and population structure are given in the Methods section. We chose 200 values of *M* between 1 and 1,000,000, and for some values of *M* we repeated the simulation 500 times in order to empirically estimate the standard errors of the estimates of ${h}_{A}^{2}$ and ${h}_{D}^{2}$. We have considered either selecting causal variants completely at random (Causal Variant Scenario A) or from only the set of variants with MAF > 0.01 (Causal Variant Scenario B). Results for Scenarios A and B are presented in Figs 1 and 2, respectively. Here, we have calculated *K* and *D* for each population as GRMs from a dense set roughly 5.8 million of frequent UK10K variants (*MAF* > 0.05). We also performed the simulation with *K* and *D* calculated on roughly 170,000 single nucleotide polymorphisms (SNPs) which are those also available in the real data of Cilento (Supplementary Figs 1 and 2).

Fitting the LMM for Isolated(1444) resulted in accurate estimates of ${h}_{A}^{2}$, estimations of ${h}_{D}^{2}$ were also unbiased but were clearly more problematic as seen by the low precision of the estimates. The results from Isolated(1444) were neither affected by the MAF range of the causal variants or the density of the genetic data used to estimate *K* and *D*. However the, precision of the estimates was low. The estimates in all of the simulated outbred populations were evidently downwardly biased when causal variants were selected completely at random and therefore included many rare variants as in the UK10K panel (from which all simulated data is based on), over 50% of the variants have a MAF below 0.01. As the size of the outbred population increases, the precision of the estimates increases but downward biases remain, even when all causal variants are non-rare. The number of causal variants for each variance component (*M*) did not affect the results other than we observed that a small number of causal variants led to lower precision in the results obtained when simulations were repeated. This is shown by the diamonds representing empirical standard errors measured for certain values of *M* shown in Figs 1 and 2 and in Supplementary Figs 1 and 2.

**Figure 3:**
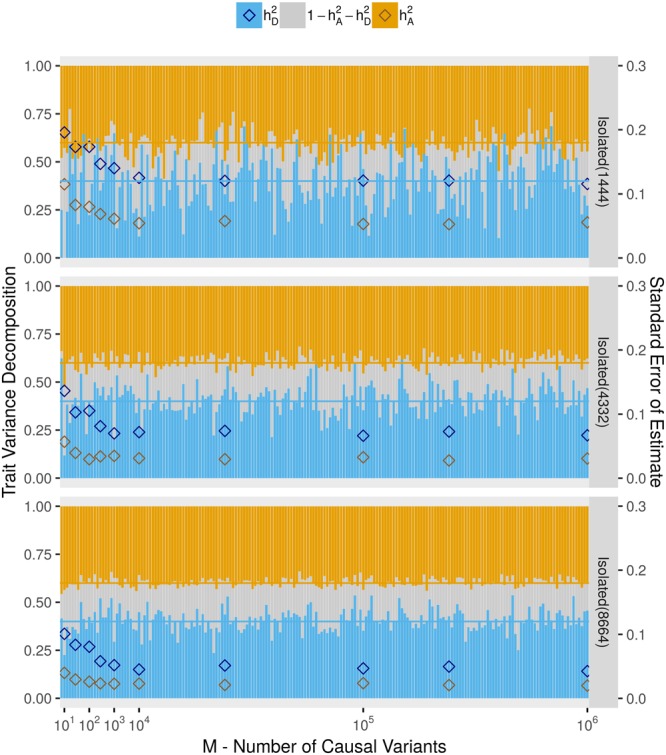
Effect of sample size on heritability estimates in an isolate. Estimates of ${h}_{A}^{2}$ and $\,{h}_{D}^{2}$ are compared for populations with isolate characteristics of size 1,444, 4,332, and 8,664. Phenotypes are simulated under Causal Variant Scenario A and under the setting ${h}_{A}^{2}=0.4$, ${h}_{D}^{2}=0.4$. Legends and the configuration of this plot are identical to those of Fig. 2.

We observed increased precision in the estimation of heritability components as we increased the size of the simulated outbred population (Figs 1 and 2). To explore the effect of sample size when studying isolates, we simulated populations with isolate characteristics of sizes 4,332 and 8,664 labelled as Isolated(4332) and Isolated(8664), respectively. A description of the simulation is given in the Methods section. For these populations, we simulated phenotypes under Causal Variant Scenarios A (displayed in Fig. 3) and B (displayed in Supplementary Fig. 3). The precisions of the estimates of ${h}_{A}^{2}$ and ${h}_{D}^{2}$ from these larger samples was increased compared to the population Isolated(1444) and estimates remained unbiased for both heritability components. Indeed, the population Isolated(8664) gave the most accurate heritability estimates of all populations thus far considered.

Subsequent analyses will focus on the population Isolated(1444). This will be of particular interest as for this population results are directly comparable with analyses of the real data of Cilento.

### Effect of the choice of relatedness matrices

To compare methods for calculating *K* and *D* in a population isolate, we performed similar simulations of phenotypes and tested the estimation of ${h}_{A}^{2}$ and ${h}_{D}^{2}$ from our LMM from each of the following strategies: *K* and *D* calculated from the pedigree of Cilento, *K* and *D* calculated from exact IBD-sharing recorded during the data simulation (true IBD), *K* and *D* calculated as GRMs, and finally *K* and *D* calculated using either the IBDLD^9^ or GIBDLD^52^ software (see Methods section). Comparisons of off-diagonal elements of these matrices are given in Supplementary Fig. 4a–d. There was clear additional variation in the true proportions of IBD-sharing as compared to the expected values calculated by the pedigree (Supplementary Fig. 4a) and this was captured by the GRMs (Supplementary Fig. 4b). The matrix *K* as estimated by a GRM was very similar to the true IBD-sharing probabilities but there were some differences for the matrix *D* (Supplementary Figure 4c). The software IBDLD and GIBDLD were able to accurately estimate the true IBD-sharing in the simulated isolate (Supplementary Fig. 4d).

**Figure 4:**
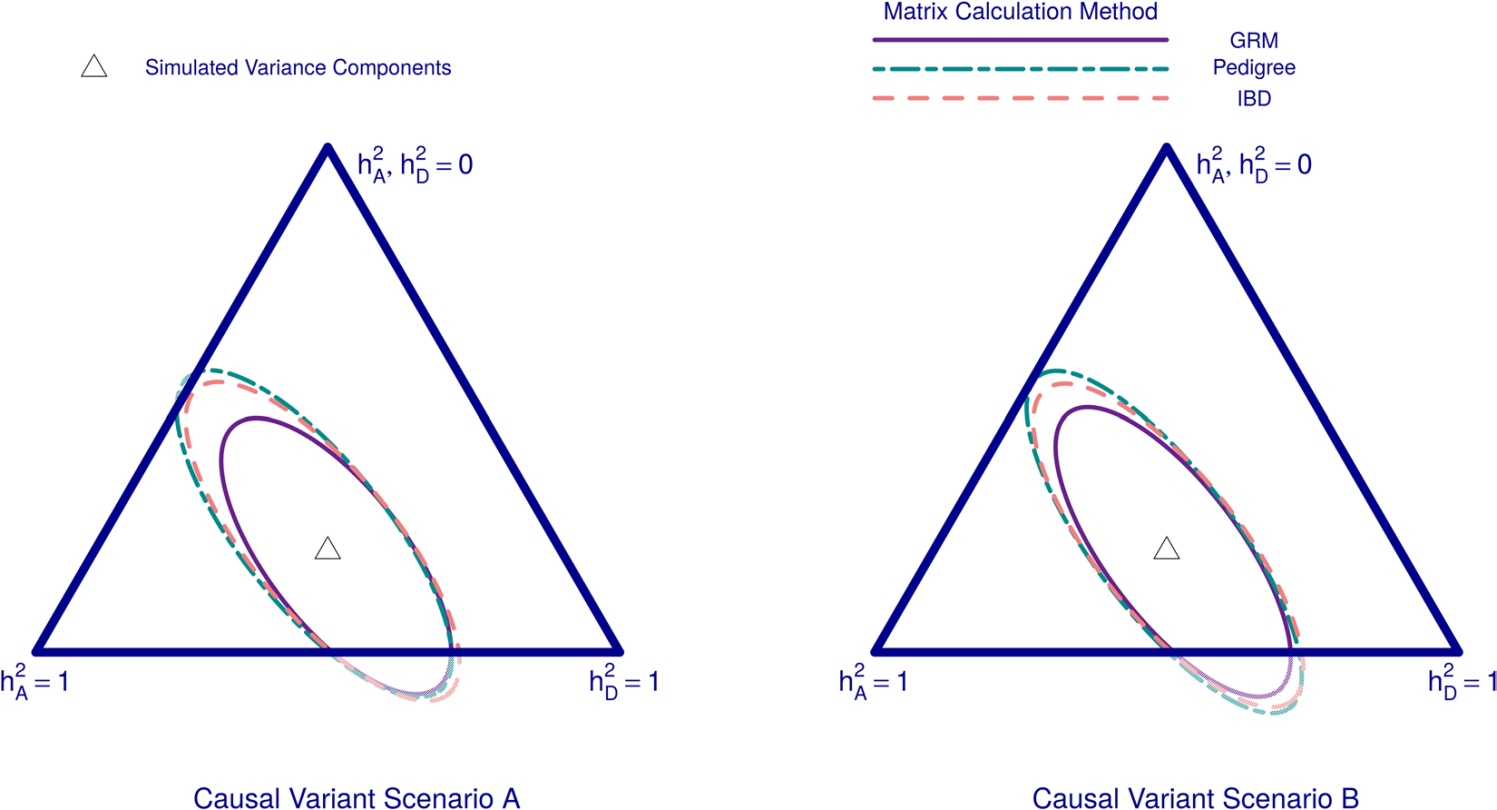
Effect of relatedness matrix estimation method in an isolate. Here, we compare methods of estimating matrices *K* and *D* for the simulated population isolate ‘Isolated(1444)’ *K* and *D* are estimated using either genetic relationship matrices (GRM), Pedigree information, or true IBD-sharing (IBD). Results are displayed on a simplex governed by the two parameters ${h}_{A}^{2}$ and $\,{h}_{D}^{2}$, which both could range between 0 and 1. The heritability scenario used to simulate all phenotypes $({h}_{A}^{2}={h}_{D}^{2}=0.4)$ is marked by the triangular point in the centre of each simplex. Minimal ellipses containing 95% of the maximum likelihood estimates (MLEs) from 500 simulated phenotypes under either Causal Variant Scenario A or B (see Figs 1 and 2) are presented. Here, phenotypes are simulated from a large set of causal variants (*M* = 100,000).

The maximum likelihood estimates (MLEs) of ${h}_{A}^{2}$ and ${h}_{D}^{2}$ from each simulated phenotype can be positioned on a simplex to represent the range of possible values of the two parameters $\,{h}_{A}^{2}$ and ${h}_{D}^{2}$. We present results from 500 simulated phenotypes with *M* = 100,000 where we display minimal ellipses that contain 95% of all MLEs obtained from each strategy (Fig. 4).

First we compare GRM estimators using roughly 5.8 million frequent (*MAF* > 0.05) UK10K positions with estimates of *K* and *D* using either pedigree information or true IBD-sharing information (Fig. 4). The method-of-moment GRM estimates appear most accurate, while true IBD-sharing based matrices performed very similarly to expected IBD-sharing matrices derived from the pedigree. This trend in results occurred irrespective of the MAFs of causal variants or the number of causal variants (Fig. 4 and Supplementary Fig. 5). The advantage observed for the GRM method is mostly evident in the estimate of ${h}_{D}^{2}$ as the ellipses were similarly sized in their minor axes (which describes variation in $\,{h}_{A}^{2}$) but more differentiable when examining their major axes (which describes variation in ${h}_{D}^{2}$). Indeed, it was on the dominance matrix *D* that we observed noticeable differences between off-diagonal elements when comparing GRMs to IBD-based methods (Supplementary Fig. 4c,d). Genomic IBD-based estimates from IBDLD or GIBDLD were also used to calculate *K* and *D*. These Hidden Markov Model (HMM) based methods are not suitable for millions of variants and so were applied to the set of roughly 170,000 SNPs present in all three Cilento villages. These methods were compared to the use of GRMs based on the same set of variants and to using pedigree information or true IBD-sharing information (Supplementary Fig. 6a,b). Such HMM methods could have improved upon the strategy using true IBD proportions as such methods could potentially uncover additional hidden IBD in our simulated population arising from IBD-sharing within the UK10K. We found that IBDLD and GIBDLD led to similar estimates of ${h}_{A}^{2}$ and ${h}_{D}^{2}$ to using either pedigree information or true IBD-sharing; and again no method was observed to outperform the use of GRMs.

### Effect of the presence of a shared environment

**Figure 5:**
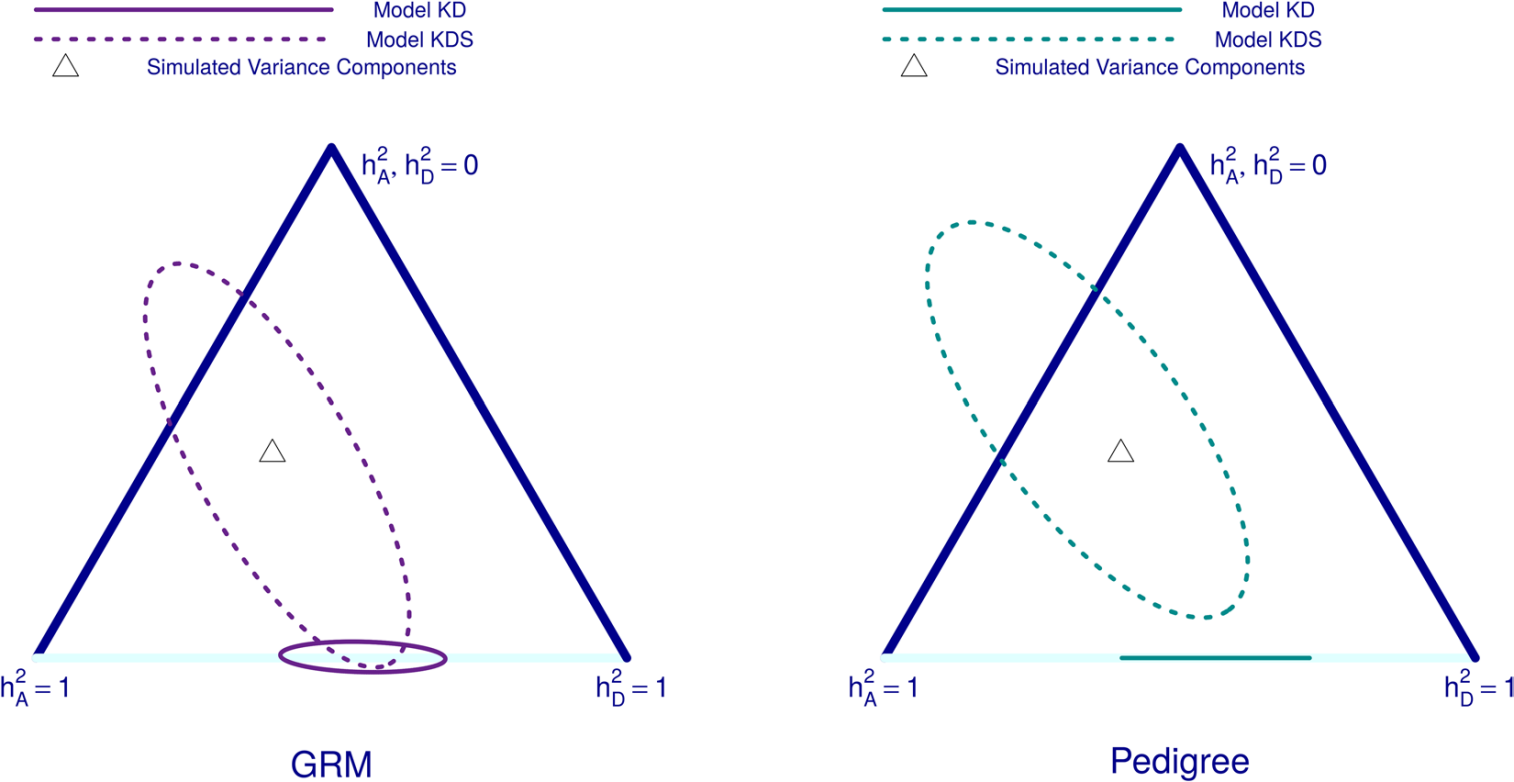
Effect of shared environmental factors on heritability component estimates in an isolate. Comparison of estimates of ${h}_{A}^{2}$ and ${h}_{D}^{2}$ under models with and without a shared environment component (model KDS and model KD, respectively). As in Fig. 4, minimal ellipses containing 95% of the maximum likelihood estimates (MLEs) from 500 simulated phenotypes but now under the setting ${h}_{A}^{2}=0.4,{h}_{D}^{2}=0.2,{h}_{S}^{2}=0.2$. Matrices *K* and *D* are calculated either using genotype relationship matrices (GRMs) or pedigree information. In the case of model KD when using pedigree information (right), all MLEs were found to be directly on the bottom edge of the simplex, and so the minimal ellipsoid degenerated into a line segment. Here, phenotypes are simulated from a large set of causal variants (*M* = 100,000).

To investigate how shared environmental factors can affect the estimation of ${h}_{D}^{2}$ in a populations isolate, we simulated additional phenotypes for the population Isolated(1444) under causal variant Scenario A, with *M* = 100,000, and with ${h}_{A}^{2}=0.4$, ${h}_{D}^{2}=0.4-{h}_{S}^{2}$, for the following values of ${h}_{S}^{2}$: 0.00, 0.02, 0.05, 0.10, 0.20, and 0.40. For each of these phenotypes, we added positive covariance between the environmental components of siblings. This covariance between siblings creates a confounding between non-additive genetic effects and shared environment effects. Full details of this phenotype simulation and the confounding created are found in the Methods section. We present the estimations of ${h}_{A}^{2}$ and ${h}_{D}^{2}$ from analyses with (model KDS) or without (model KD) the inclusion of a variance component (*S*) indicating pairs of siblings in the sample for ${h}_{S}^{2}=0.20$ (Fig. 5). Throughout, model names indicate the set of variance-covariance matrices included in the LMM. Results for further values of ${h}_{S}^{2}$ are displayed in Supplementary Fig. 7a–f. Here, we used either GRMs or pedigree based estimates for *K* and *D* as these were predominantly the methods used in aforementioned studies that calculated dominant genetic components for widely studies traits (Table 1). Our simulations indicate that once a significant correlation between siblings is introduced, our unadjusted estimates for the broad-sense heritability became close to or equal to 1 (MLEs falling on the bottom axis of the simplex for model KD). Again, in these analyses using GRMs appears to outperform the use of pedigree based estimates. Adjusting for such correlation between siblings in the LMM did substantially correct for this bias but it is clear that in a population such as Cilento, there is little hope in effectively discriminating between dominant genetic variability and shared environmental factors between siblings if both occur simultaneously.

An obvious approach to avoid such ambiguity would be to remove one individual from every pair of siblings but in Cilento this would greatly reduce the sample size. Therefore, we removed one individual from each pair of siblings from the population Isolated(8664), creating a sibling free population which we label as “Isolated(5136)_nosibs”. Full details of the simulation of this population are found in the Methods section. From this population, we observed improved estimates of both ${h}_{A}^{2}$ and ${h}_{D}^{2}$ as compared to the Outbred(8664) under Causal Variant Scenario A; with the two populations performing similarly under Causal Variant Scenario B (Supplementary Fig. 8a,b). When compared to the results from Isolated(1444), the absence of pairs of individuals with high IBD = 2 probabilities led to a slight underestimation of ${h}_{D}^{2}$, but the increased sample size led to lower standard errors across replications of phenotype simulation. If no dominant genetic component was simulated, the Isolated(1444) population was most likely to give large (more erroneous) estimates for ${h}_{D}^{2}$ compared to Isolated(5136)_nosibs and Outbred(8664) (Supplementary Fig. 8c,d).

### Analysis of the Cilento Isolates

We first calculated the matrices *K* and *D* using different approaches and then compared the resulting values. We calculated *K* and *D* using either the pedigree information, or as GRMs using genotype data before or after imputation. Results were in accordance with those from the simulated population isolate Isolated(1444) (Supplementary Fig. 9). However, we observed greater differences between the off-diagonal elements calculated with the pedigree and those in the GRMs when analysing the real Cilento data as compared to Isolated(1444). This is likely to stem from the explicit use of the pedigree information within the simulation. The inclusion of imputed variants led to similar estimates for the matrices *K* and *D* (Supplementary Fig. 10).

**Figure 6:**
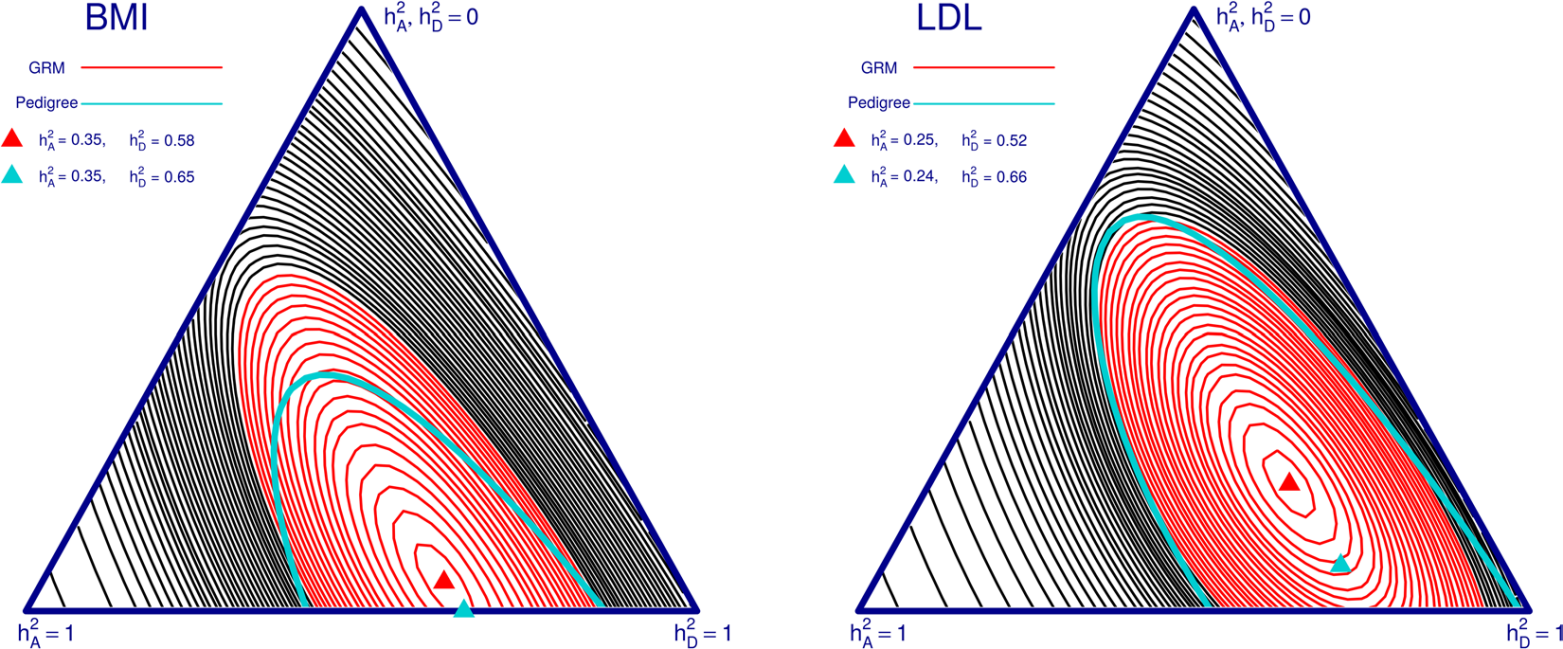
Heritability analysis for BMI and LDL in Cilento. Black contours represent the likelihood profile from the model KD (see Fig. 5), with matrices *K* and *D* calculated as genetic relationship matrices (GRMs). The red zone represents the 95% confidence interval for the red maximum likelihood estimate (MLE) (red triangular peak). The corresponding MLE and 95% confidence boundary for the analysis using pedigree information to estimate *K* and *D* are added to the plot in blue.

Following quality control and imputation (full details are given in the Supplementary Materials); we fitted LMMs to the data in Cilento having estimated matrices *K* and *D* as GRMs (using all variants with MAF > 0.05 and imputation quality score > 0.7). Several traits displayed significant dominant genetic components and our results (Table 2) are not distant to those found in the literature of previous studies in population isolates (Table 1). LMMs were fitted with different combinations of the matrices *K*, *D*, and *S* (the sibling indicator matrix). Full details are given in the Methods section; as above in the simulation study, the model names indicate the variance components included in the LMM. The orthogonality between the additive and non-additive genetic components is apparent as estimates for ${h}_{A}^{2}$ are similar across models with or without the inclusion of the non-additive genetic variance component. For each phenotype considered, we estimated the entire likelihood surface as well as the MLEs for the parameters ${h}_{A}^{2}$ and ${h}_{D}^{2}$ under the model KD. Likelihood surfaces governed by ${h}_{A}^{2}$ and ${h}_{D}^{2}$ for BMI and LDL are displayed in Fig. 6 and corresponding results for other traits are found in Supplementary Fig. 11a–d. We observed similar profiles in the likelihood contours as were observed in the distributions of MLEs from repeated phenotype simulation in the simulation study. We are able to have a reasonable level of confidence in the estimates of the additive genetic component, but the dominant genetic component is problematic as our confidence regions are very wide. The MLEs found when using pedigree information to estimate matrices *K* and *D* had equivalent estimates for the additive genetic components to the MLEs found when using GRMs, however the dominant genetic components were always estimated as equal or greater when using pedigree information.

**Table 2:** Maximum likelihood estimates for the contribution of each variance components considered in a Linear Mixed Model (LMM).

Phenotype	GRM Model: K	GRM Model: KD		GRM Model: KS		GRM Model: KDS			Pedigree Model: K	Pedigree Model: KD		Pedigree Model: KS		Pedigree Model: KDS		
	${{\boldsymbol{h}}}_{{\boldsymbol{A}}}^{{\bf{2}}}$	${{\boldsymbol{h}}}_{{\boldsymbol{A}}}^{{\bf{2}}}$	${{\boldsymbol{h}}}_{{\boldsymbol{D}}}^{{\bf{2}}}$	${{\boldsymbol{h}}}_{{\boldsymbol{A}}}^{{\bf{2}}}$	${{\boldsymbol{h}}}_{{\boldsymbol{S}}}^{{\bf{2}}}$	${{\boldsymbol{h}}}_{{\boldsymbol{A}}}^{{\bf{2}}}$	${{\boldsymbol{h}}}_{{\boldsymbol{D}}}^{{\bf{2}}}$	${{\boldsymbol{h}}}_{{\boldsymbol{S}}}^{{\bf{2}}}$	${{\boldsymbol{h}}}_{{\boldsymbol{A}}}^{{\bf{2}}}$	${{\boldsymbol{h}}}_{{\boldsymbol{A}}}^{{\bf{2}}}$	${{\boldsymbol{h}}}_{{\boldsymbol{D}}}^{{\bf{2}}}$	${{\boldsymbol{h}}}_{{\boldsymbol{A}}}^{{\bf{2}}}$	${{\boldsymbol{h}}}_{{\boldsymbol{S}}}^{{\bf{2}}}$	${{\boldsymbol{h}}}_{{\boldsymbol{A}}}^{{\bf{2}}}$	${{\boldsymbol{h}}}_{{\boldsymbol{D}}}^{{\bf{2}}}$	${{\boldsymbol{h}}}_{{\boldsymbol{S}}}^{{\bf{2}}}$
Height	0.76	0.74	0.13	0.74	0.04	0.74	0.12	0.01	0.75	0.74	0.15	0.74	0.04	0.74	0.15	0.00
BMI	0.40	0.35	0.58	0.31	0.23	0.31	0.00	0.23	0.44	0.35	0.65	0.35	0.21	0.35	0.00	0.21
TGLY	0.27	0.24	0.26	0.21	0.11	0.21	0.00	0.11	0.28	0.23	0.45	0.23	0.11	0.23	0.41	0.01
HDL	0.49	0.49	0.00	0.44	0.02	0.44	0.00	0.02	0.48	0.49	0.00	0.48	0.01	0.48	0.00	0.01
Total Chol	0.29	0.23	0.55	0.23	0.18	0.22	0.27	0.12	0.29	0.21	0.72	0.22	0.18	0.21	0.47	0.06
LDL	0.32	0.25	0.52	0.24	0.17	0.23	0.29	0.10	0.33	0.24	0.66	0.24	0.16	0.24	0.45	0.06

Model names refer to the set of variance components included. *K* denotes the additive genetic component, *D* the non-additive or dominant genetic component, and *S* the component accounting for shared environmental effects between siblings. The previously reported results from Table 1 can be compared to our results under the model KD. Matrices *K* and *D* are calculated either as genetic relationship matrices (GRMs) or from pedigree information.

The traits of BMI, LDL, and Total Chol were all estimated as having dominant genetic components higher than their respective additive genetic components in the KD model. By examining the 95% confidence regions, there is some indication that the dominant genetic components are unlikely to be equal to zero. This is due to the observation that the red zones either do not intersect or only briefly intersect the upper left boundary $({h}_{D}^{2}=0)$ of their respective simplexes (Fig. 6 and Supplementary Fig. 11d).

**Figure 7:**
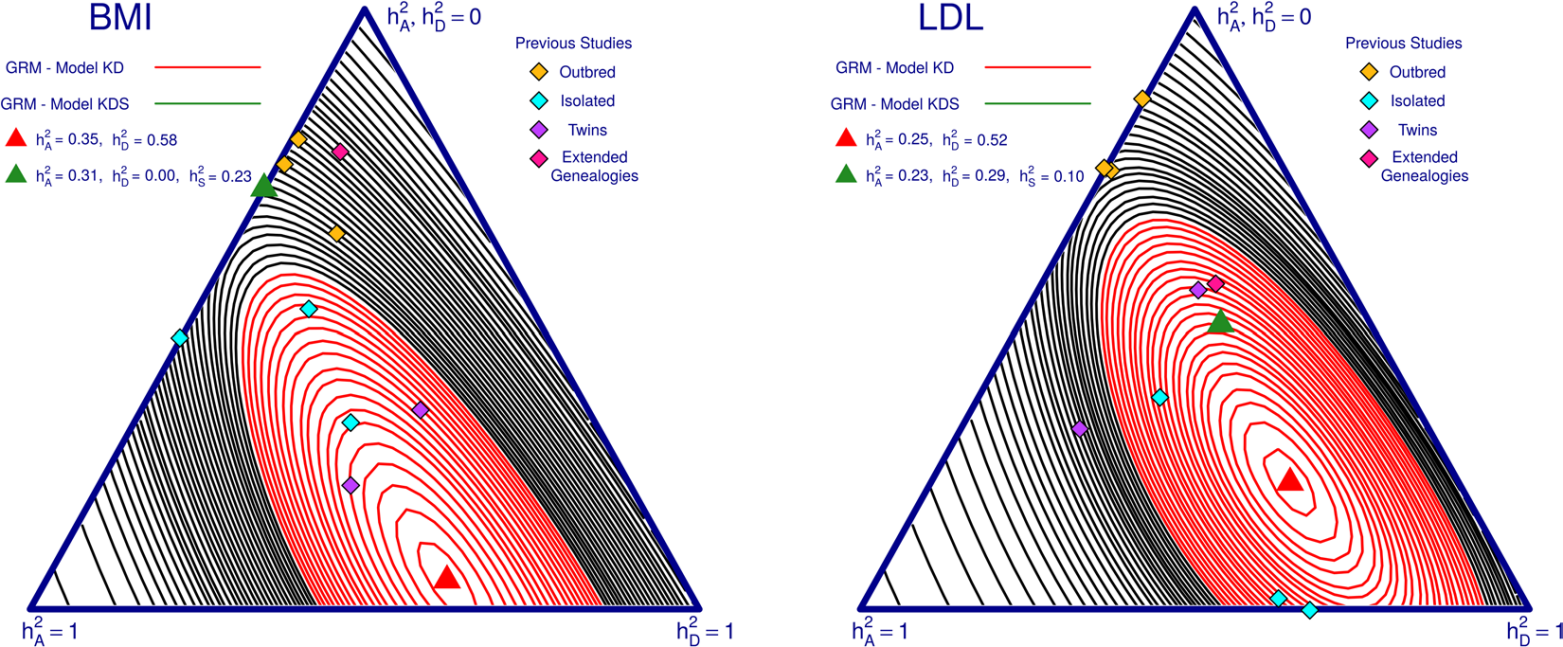
Effect of shared environmental factors on heritability analysis for BMI and LDL in Cilento. Here we compare models KD and KDS (see Fig. 5) for the two traits in Cilento. Black contours represent the likelihood profile for the model KD, with the red zone indicating the 95% confidence interval for the red maximum likelihood estimate (MLE) (red triangular peak). The corresponding MLE for the KDS model is added in green. We also add in the previously observed estimates from the literature (Table 1).

Adding the shared environmental component between siblings drastically changed the estimates of ${h}_{D}^{2}$ for many traits as seen by comparing models KD and KDS in Table 2; for our two example traits (BMI and LDL) we present again the likelihood profiles from the original analysis and then new MLE and 95% confidence interval for ${h}_{A}^{2}$ and ${h}_{D}^{2}$ from the KDS model as well as the previous estimates for ${h}_{A}^{2}$ and ${h}_{D}^{2}$ found in the literature (Fig. 7). Equivalent plots for our other studied traits are given in Supplementary Fig. 12a–d.

For BMI, the unadjusted heritability estimate was distant from previously reported results, but once we allow for a shared environmental component between siblings, we find similar estimates for ${h}_{D}^{2}$ to previous studies. For LDL, the unadjusted heritability estimates lay close to previous results from isolated populations, with the adjusted results moving towards previous results in studies of outbred populations but remaining quite large at 0.29.

## Discussion

Across all analyses, whether on simulated or real Cilento data, we observed that estimates of ${h}_{D}^{2}$ had less precision than estimates of ${h}_{A}^{2}$.

Isolated populations exhibit favourable characteristics for uncovering the contribution of ${h}_{D}^{2}$ due to the increased proportions of IBD = 2 between individuals. Our simulation elaborates on this by showing that in the absence of shared environmental effects, estimating ${h}_{D}^{2}$ (and indeed ${h}_{A}^{2}$) from an LMM in a population isolate will yield unbiased results for polygenic phenotypes with wide a range of characteristics. However, we saw that shared environmental factors pose a non-trivial obstacle to analysing dominant genetic variance of a trait in an isolated population. In the presence of even small shared environmental effects between siblings in the simulated isolate, we observed that estimates of ${h}_{D}^{2}$ are heavily biased. Improved estimates may be attainable by including a sibship matrix in the variance decomposition analysis but accurately partitioning between dominance effects and shared environmental effects through linear mixed modelling in a population such as Cilento may not be possible.

We compared different methods to estimate the covariance matrices *K* and *D*. In the simulated isolate, the precision of the estimates of ${h}_{D}^{2}$ was either larger or equivalent when using GRMs as compared to IBD-based methods. This had previously also been noted by Browning & Browning^53^ when estimating ${h}_{A}^{2}$. Furthermore, it would appear that only a relatively small number of SNPs are required to compute such GRMs in an isolate as using far denser sets of variants (either in our simulation or through imputation in the Cilento dataset) did not noticeably affect the fitting of the LMM. The advantage observed for GRMs could be because they can capture similarities between all types of pairs of individuals in the isolate; including similarities not described by the recorded pedigree structure or originating before the founding event of the population. Therefore this approach combines the classical interpretation of heritability regarding closely related individuals with the more recent approaches involving samples of unrelated individuals.

Foreseeably, the simulated outbred populations led to underestimation of both ${h}_{A}^{2}$ and ${h}_{D}^{2}$ in most of the settings of phenotype simulation. This may go some way to explain the differences between estimates of ${h}_{D}^{2}$ that we observed in the literature for many complex traits. Our results suggest that observing very different estimations for non-additive genetic components between isolates and outbred populations could indicate the presence of many causal variants that occur at low frequencies across populations and that have non-zero dominant genetic effects. However, such an observation could also indicate the presence of bias due to the shared environmental factors in the studies of isolates. We note that estimation from outbred populations can also suffer from biases arising from shared environmental factors due to hidden structures existing within the population; a scenario that we have not considered in our simulation study. Population stratification within a cohort is a known example of a structure that can lead to bias in heritability studies of unrelated individuals^54,55^.

The heritability analyses that we have carried out in Cilento did indeed suggest the presence of non-additive genetic variance for some of the traits considered. However, the phenotypes studied in Cilento behaved in similar ways to the simulated phenotypes with added non-genetic correlation between siblings. The simulation study suggested that even a very small shared environmental effect between siblings could result in the disparate heritability estimates we observed in Cilento between fitting LMMs with and without a variance component for covariance between siblings. When the simulated shared environmental component was large, broad-sense heritability estimates approached 1; this is a result we observed in both previous studies of isolates for many traits^38,39^ (see Table 1) and in Cilento for the trait BMI. Combining this observation with the wide observed ranges of estimates for ${h}_{D}^{2}$ in the literature strongly suggests that previous results in isolates have thus far been inflated by shared environmental effects and that ${h}_{D}^{2}$ statistics have been overestimated. For a trait such as LDL, we still observed high estimates for ${h}_{D}^{2}$ even when accounting for a shared environment effect in the model, a result which our simulation suggests would be unlikely if indeed ${h}_{D}^{2}=0$ for this trait.

It has been argued that the classical separation of the two additive and non-additive genetic components may lead to higher estimates for the additive genetic variance over the non-additive genetic variance^56^. However, proposed alternative definitions are far less interpretable and lead to variance decompositions with less applicable value. Higher order non-additive genetic variance components could be contributing to our estimates of dominance in Cilento^5^. Indeed, we recognise that ignoring the presence of epistatic effects has been shown to lead to overestimations of *H*^2^ by Zuk *et al*.^57^ who also proposed a non-parametric method for estimating heritability in a population isolate. Such approaches require large samples of pairs of individuals with identical expected relatedness coefficients. Similar approaches include those based on Haseman-Elston regression^58^ and studies focusing on populations of siblings or nuclear families. However, for the data of Cilento such methods proved not to be applicable due to the variety of relationships between pairs, such that looking at each pair type separately resulted in sample sizes too small to provide realistic estimations. There exist a wide range of sophisticated approaches for calculating narrow-sense heritability in sample of unrelated individuals^59–61^. Zaitlen *et al*.^41^ proposed to dissect narrow-sense heritability in samples containing close relatives by splitting variance between GRMs and thresholded GRMs, and isolated populations could prove a valuable resource for future studies using such approaches. However, as we include non-additive genetic components and wish to compare our results to studies using pedigree based methods, we have not explored such concepts here.

In this study, we have demonstrated various phenomena which can either result in under-estimation of ${h}_{D}^{2}$ in studies of outbred populations or over-estimation in studies including closely related individuals. At this juncture, the existence of significant non-zero dominant genetic variation for many traits remains uncertain, but this could be elucidated through the continued gathering of estimates from diverse populations. Whilst different populations harbour differing levels of environmental variation, and hence one cannot expect agreement on heritability estimations, studies of isolated populations could lead to more reliable conclusions as to the existence or non-existence of genetic dominance for complex traits. If significant estimates for ${h}_{D}^{2}$ are found when accounting for a shared environment effect between siblings, this is indicative of a true non-zero dominance component.

One possible future direction would be to increase the sample size in a study of an isolate. However, as this will not usually be feasible for a single isolate, one strategy that could be particularly interesting would be to combine data from several isolates with similar ancestral origins. Such an approach could give high precisions of the estimates of both ${h}_{A}^{2}$ and ${h}_{D}^{2}$ due to the large sample size. Importantly, this strategy could also provide a large enough sample to complete analyses without sibling pairs, and to facilitate appropriate sensitivity analyses regarding the presence of siblings.

## Methods

### The Cilento Isolate

The Cilento isolate comprise three villages from the South of Italy; Campora, Cardile, and Gioi. Pedigree, phenotypic, and genetic data have previously been gathered as part of the Cilento Study. A pedigree structures which connects all three village has been reconstructed from parish records. The three villages have been shown to represent characteristics of population isolates intermediate between the large isolate population of Iceland^62^ and the highly isolated Hutterite population^63,64^. Aggregating over the three villages, we have a pedigree of 7,585 members including 1,444 genotyped members. The high quality of the reconstructed genealogy in Cilento makes it an appropriate tool for simulating a realistic example of data from an isolated population. Individuals from Campora and Cardile have been genotyped on an Illumina 370 K array, whilst individuals from Gioi have been genotyped on an Illumina HumanOmniExpress array. Deep phenotyping has been performed in Cilento for a range of anthropometric, cardiometabolic, and haematological traits. For the purposes of this study, we have concentrated on phenotypes that have been often analyzed in the literature of both other population isolates and in samples of unrelated individuals (Supplementary Table 1).

### Simulation of genotypes and phenotypes

To create simulated datasets, we created mosaic haplotypes using the same stochastic recombination model as in the generation of control individuals by the software HapGen2^65^. We took the UK10K imputation panel as reference haplotypes having first removed one individual from every pair of twins present in the panel. To simulate unrelated individuals we sampled 22 pairs of mosaic chromosomes, where each section of their mosaics is copied from a randomly sampled haplotype from the pool of UK10K haplotypes. In this manner, we created a sample of 8,664 (6 × 1,444) unrelated individuals. To create isolate type data, for each chromosome, we randomly selected 200 UK10K haplotypes, from which 2,940 mosaic haplotypes were simulated in order to simulate the 1,470 founders of the combined pedigree of Cilento. This set of founder haplotypes were supplied to the software Genedrop (part of the MORGAN^66^ package) along with the pedigree of Cilento in order to simulate phased genetic data for the 1,444 genotyped members of Cilento. Our gene-dropping approach was identical to the methods used in Herzig *et al*.^67^ We have made comparisons on four potential populations: the 1,444 individuals from Genedrop with isolate type data, labelled “Isolated(1444)”, and three possible sets of the 8,664 simulated unrelated individuals: “Outbred(1444)”, “Outbred(4332)”, “Outbred(8664)”, that represent outbred populations of the same size as Cilento, three times the size, and six times the size, respectively. We chose this range of samples sizes based on an analysis of the variance of eigenvalues^68^ of GRMs estimated on the populations Isolated(1444) and Outbred(1444) (Supplementary Materials and Supplementary Table 2). The choice of 200 haplotypes for the generation of founder haplotypes for Cilento stems from the previous work which estimated that 96.7% of the genetic diversity in Campora is accounted for by 17 female and 20 male lineages^63^. This would suggest that 74 (37 × 2) autosomal haplotypes would be appropriate for the generation of simulated data for Campora and we decided to scale this up to 200 for the generation of simulated data for the three villages. We checked that this created simulated data with a similar structure as the observed data in Cilento (Supplementary Table 2 and Supplementary Fig. 13).

Our method for simulating isolate-type data requires a pedigree for gene-dropping. To create larger datasets with isolate characteristics, we used the Cilento pedigree multiple times. In detail, we simulated six populations of size 1,444, each using the Cilento pedigree but with different random draws of founding haplotypes. We then combined the first three and all six of these populations to create the populations Isolated(4332) and Isolated(8664), respectively. In addition, we randomly discarded one individual from each sibling pair of the population Isolate(8664) to create a population with no sibling pairs of size 5,136, labelled as “Isolated(5136)_nosibs”.

Phenotypes were simulated repeatedly for each population as the sum of normally distributed errors (Equation 3).

3 $$Y={\beta }_{A}^{T}{G}_{A}+{\beta }_{D}^{T}{G}_{D}+\varepsilon $$

*G*_*A*_ and *G*_*D*_ are the additive genetic components of the genotypes of the randomly selected *M* causal additive variants and the non-additive genetic components of the randomly selected *M* causal dominant variants, respectively. Effect sizes *β*_*A*_ and *β*_*D*_ were drawn from normal distributions. Variants may exhibit both additive and dominant effects and a maximum of 2*M* variants could have non-zero effect sizes. We varied the heritability by scaling the effect-sizes accordingly in the knowledge that ${\tau }_{A}=\sum {\beta }_{A}^{2}$ and ${\tau }_{D}=\sum {\beta }_{D}^{2}$. We have simulated a range of possible phenotype characteristics by varying the number of causal variants and the MAFs of causal variants.

To estimate the variance parameters, and hence heritability, we fitted the model of Equation 1 in the R-package ‘Gaston’^69^ and estimated parameters *τ*_*A*_, *τ*_*D*_, and ${\sigma }_{E}^{2}$ using Average Information Restricted Maximum Likelihood Estimation (AIREML)^70^. Matrices *K* and *D* were estimated using the method-of-moment techniques described in Zhu *et al*.^12^, and we either used all variants present on the UK10K, or the variants present in the real data from all three Cilento villages. The exact set of variants used for these calculations were those with MAF > 0.05 and those passing a quality control threshold on the Hardy-Weinberg p-values (>10^−5^).

In the case of Isolated(1444), we also estimated *K* and *D* from the pedigree structure of Cilento using software IdCoefs^4^ that calculates ${{\rm{\Delta }}}_{1},\ldots ,{{\rm{\Delta }}}_{9}$ through the recursive algorithm described by Karigl^71^. Furthermore, we were able to record the origin of every mosaic segment simulated during the HapGen based and gene-dropping stages. This allowed us to calculate true proportions of IBD-sharing between every pair of individuals in the Isolated(1444) population. We also tested the software IBDLD^9^ and GIBDLD^52^ which directly estimate ${{\rm{\Delta }}}_{1},\ldots ,{{\rm{\Delta }}}_{9}$. For IBDLD, we used the LD-RR mode, default parameters, and we supplied the software with the expected values of ${{\rm{\Delta }}}_{1},\ldots ,{{\rm{\Delta }}}_{9}$ between all pairs from the pedigree (calculated by IdCoefs). Conversely, GIBDLD used only the genotypes; we also ran this software with default parameters. For both IBDLD and GIBDLD, we used only the SNPs present in both genotyping arrays in Cilento as the software were not designed for sequence data.

Here we introduce the sibship matrix, denoted as *S*, which has values of 1 on the diagonal and at every off-diagonal element corresponding to pairs of siblings in the sample; all other entries are zero. To simulate phenotypes for the population Isolated(1444) with additional correlation between pairs of siblings, approximating an effect of shared environmental exposure, we simulated phenotypes under the same model as Equation 3 except that the environmental components were no longer drawn independently from normal distributions, but from a multi-variate normal distribution with zero mean and a covariance structure of $({\sigma }_{E}^{2}+{\sigma }_{S}^{2}){I}_{N}+{\sigma }_{S}^{2}S$; a matrix with ${\sigma }_{E}^{2}+{\sigma }_{S}^{2}$ on the diagonal and ${\sigma }_{S}^{2}$ on every off-diagonal entry corresponding to a pair of siblings in the sample. We chose values of ${\sigma }_{S}^{2}$ in order to create phenotypes with ${h}_{S}^{2}$: 0.00, 0.02, 0.05, 0.10, 0.20, and 0.40 where ${h}_{S}^{2}={\sigma }_{S}^{2}/({\tau }_{A}+{\tau }_{D}+{\sigma }_{S}^{2}+{\sigma }_{E}^{2})$.

### Analysis of Cilento Data

After quality control on both phenotypes and genetic data (details in the Supplementary Materials), we used the same approach as with the simulated data to estimate the heritabilities of the seven traits considered in this study. The only difference being that for the analyses of Cilento data, we added the following covariates to the LMM: age, sex, age × sex, and indicators of village membership (Campora, Cardile, or Gioi). For one trait (Triglycerides) we transformed the phenotype to a logarithmic scale, whereas other traits were left untransformed after excluding very small numbers of outliers. LDL and Total Chol were both pre-adjusted for use of lipid-lowering medication. Matrices *K* and *D* were again estimated on the basis of pedigree or genetic information. To calculate GRMs from genetic data, we were restricted to using the set of variants on the intersections of the two arrays used for genotyping of Cilento data. As this set was relatively sparse, we also performed genetic imputation with the following pipeline: phasing by SHAPEIT2^72^ with the “duohmm” option^73^ and informed by the Haplotype Reference Consortium^74^ (HRC) reference panel followed by imputation by IMPUTE4^75^ with the HRC as the reference panel. *K* and *D* were then computed on hard called imputed genotypes^76,77^ after removing variants with imputation quality scores below 0.7.

In a recent study of the Icelandic population, Young *et al*.^78^ presented an IBD-based method for nuclear families in the Icelandic population aimed at eliminating environmental bias by looking at deviations in observed kinship from expected values. In Cilento data, the sample size precluded this approach as there are insufficient numbers of pairs of individuals with the required expected level of IBD-sharing and with both sets of parent’s genotypes. However, we are able to add a shared environment effect by adding into our model a variance component indicating pairs of individuals who share the same mother. A similar approach was shown to lead to unbiased results in many simulation settings in Young *et al*.^78^ As pairs of siblings have by far the highest probability of sharing two alleles IBD as each locus (one chance in four), correlations caused by shared environmental exposures between siblings are very likely to confound the estimation of ${h}_{D}^{2}$. If there is significant confounding, this should be indicated by a large difference in results when including such a matrix indicating siblings in the LMM. We fitted four LMMs for every trait which we denote as model K, model KD, model KS, and model KDS to indicate the set of variance-covariance matrices included in the model.

## Electronic supplementary material

Supplementary Materials

## Data Availability

The UK10K panel of haplotypes is available from the European Genome-phenome Archive and the simulation scripts are available from Anthony Francis Herzig (anthony.herzig@inserm.fr) on reasonable request. The Cilento datasets analysed during the current study are available from Marina Ciullo (marina.ciullo@igb.cnr.it) on reasonable request and on a collaborative basis.
