# Supplementary material for: Detecting the dominance component of heritability in isolated and outbred human populations
Source: Sci Rep. 2018 Dec 21;8:18048. doi: 10.1038/s41598-018-36050-7 (PMC6303332; doi:10.1038/s41598-018-36050-7)
Supplement: Supplementary file 1 — Supplementary Materials [file 41598_2018_36050_MOESM1_ESM.pdf]

Detecting the dominance component of heritability in isolated and outbred human populations

Anthony F. Herzig[1,2,†], Teresa Nutile[3], Daniela Ruggiero[3,4], Marina Ciullo[3,4,†], Hervé Perdry[5,‡], Anne-Louise Leutenegger[1,2,‡]

1. *Inserm, U946, Genetic variation and Human diseases, F-75010 Paris, France*
2. *Université Paris-Diderot, Sorbonne Paris Cité, U946, F-75010 Paris, France*
3. *Institute of Genetics and Biophysics A. Buzzati-Traverso - CNR, Naples, Italy*
4. *IRCCS Neuromed, Pozzilli, Isernia, Italy*
5. *Université Paris-Saclay, Univ. Paris-Sud, Inserm, CESP, Villejuif, France*

† *Corresponding authors*

‡ *Co-senior authors*

Supplementary Materials

Quality Control

Using the known pedigree structure of the Cilento isolates, we scanned for Mendelian errors within the genotype data using Plink [76] and set all genotypes to missing within nuclear families wherever such errors were found. We then removed eight individuals due to very high levels of missingness (over 5%) and restricted to the set of shared SNPs between the two genotyping arrays used in Cilento. Finally we removed variants with minor allele frequencies less than 0.01, Hardy-Weinberg p-values less than 10^{-5} and with missingness greater than 5%. This left 173,911 SNPs from which to calculate GRMs for subsequent heritability analysis. Finally, it became apparent that three pairs of monozygotic twins were present within the sample. We decided to remove one member at random from each pair from subsequent heritability analysis. These twin pairs can be observed in Supplementary Figures 9 and 10.

In Supplementary Table 1 we detail the seven phenotypes studied here, for each phenotype we removed values lying more than three standard deviations away from the observed mean (after transformations (if any) had been applied.

Imputation

Phasing and imputation were completed separately on the two sets of individuals coming from different genotyping arrays in Cilento. We reconstructed genetic phase in Cilento from SHAPEIT2 [69] with the 'duohmm' option [70]. SHAPEIT2 was employed with 15 burn-in iterations, 15 pruning iterations, 35 main iterations and we used a reduced version of the HRC panel [71] to inform phasing. Following this, we performed haplotype imputation using IMPUTE4 [72] and the same version of the HRC panel. This reduced HRC panel used here included 27,165 individuals and was made available to us from the European phenome-genome archive. IMPUTE4 was applied using default parameters in windows of 5Mb with 250Kb buffer regions. Imputation quality scores ('info') were calculated with the software QCTOOL.

Following imputation, we removed all variants with an 'info' score less than 0.7 in either of the two genotyping arrays and called most likely genotypes whenever an individual genotype had a posterior probability greater than 0.9. Otherwise, imputed genotypes were set to missing. This led to a final count of 3,757,339 confidently imputed variants.

Simulation

Our HapGen [64] like haplotype mosaic simulated was tuned to produce mosaic pieces of average size of 1-2cM, both in the case of creating simulated populations of outbred individuals, and when creating founding haplotypes for gene-dropping onto the Cilento pedigree. Here we set the effective population size to 3000 and we chose not to simulate mutations, genotyping errors, or missingness in our datasets. The mosaicism was tuned in order to achieve similar kinship and structure in the simulated isolated population as observed in the observed data in Cilento. Indeed, in Supplemental Table 2 we give the variances of the eigenvalues of all GRM matrices calculated both on the Cilento dataset studies here as well as the various simulated datasets and in Supplementary Figure 13 we compare a simple principle component analysis in Cilento and in the simulated population isolate

based on the pedigree of Cilento - Isolated(1444). Through these analyses, we observe that our simulation of an isolate appears to have successfully created similar structure to the Cilento data.

It can be shown that the precision of the estimate of a variance parameter in a linear mixed model is proportional to the product of the sample size and the variance of eigenvalues of the associated variance-covariance matrix [66]. Hence, as we observed that the variances of eigenvalues of the matrix K in the Outbred(1444) were roughly 36 times smaller than the corresponding values in Isolated(1444), we reasoned that an outbred population of 6×1444 (8664) individuals would approximately give us equivalent precision to the population Isolated(1444). Thus we simulated an Outbred population of size 8664, as well as an intermediate population of size 3×1444 (4332) in order to observe any trends in the results relating to sample size.

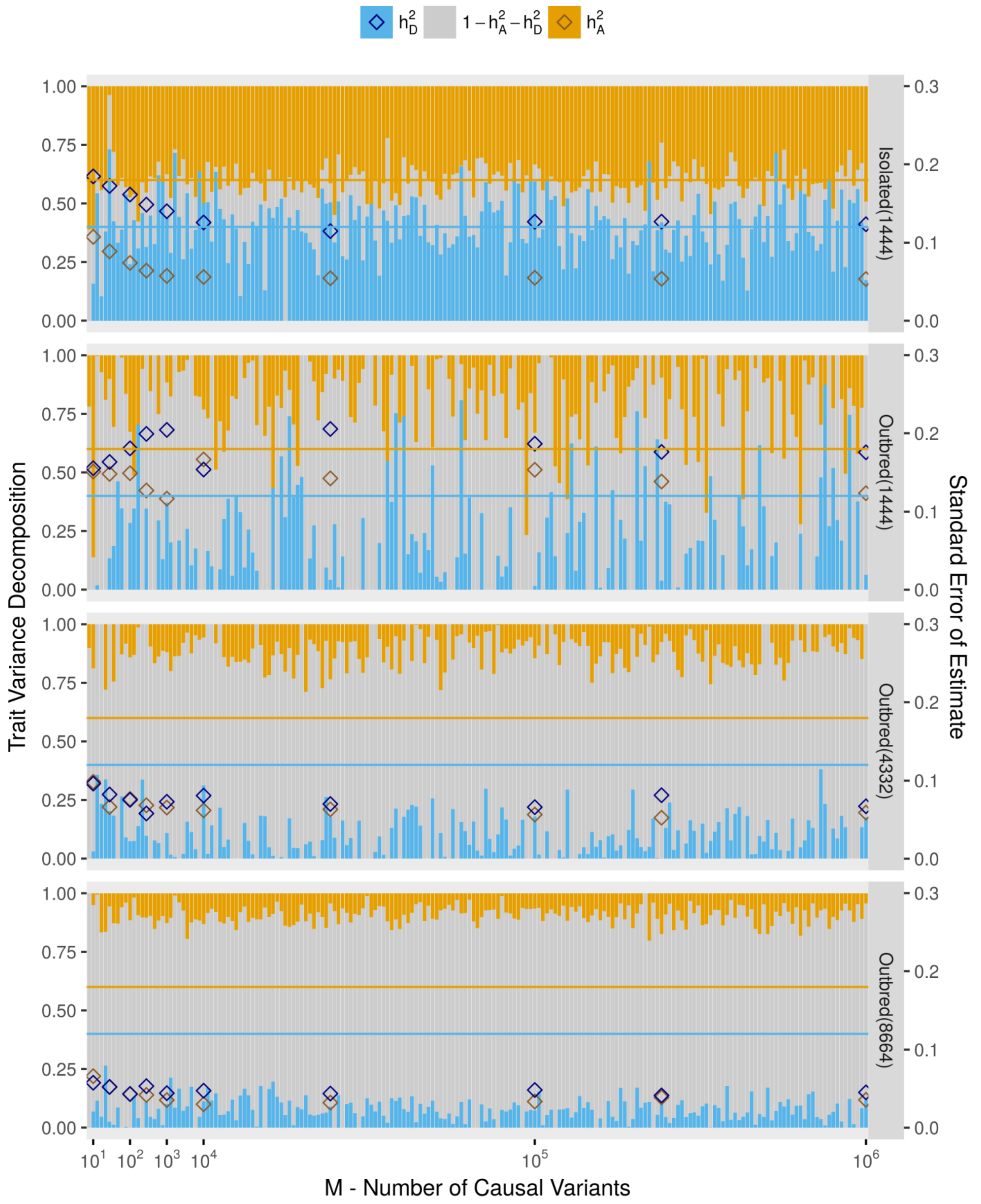

Supplementary Figure 1.

Estimates of h_A^2 and h_D^2 are represented for each simulated phenotype. Each phenotype was simulated using different numbers of causal variants (M) for each variance component. Results from four simulated populations are given, either Isolated(N) or Outbred(N), where the value of N denotes the sample size.

Matrices K and D are calculated using roughly 170,000 variants present in all villages in Cilento, causal variants are selected completely at random (Causal Variant Scenario A).

Diamonds represent empirical standard errors measured for certain values of M which are measured on the right vertical axes. A missing bar for h_A^2 or h_D^2 indicates the maximum likelihood estimate of the parameter was zero.

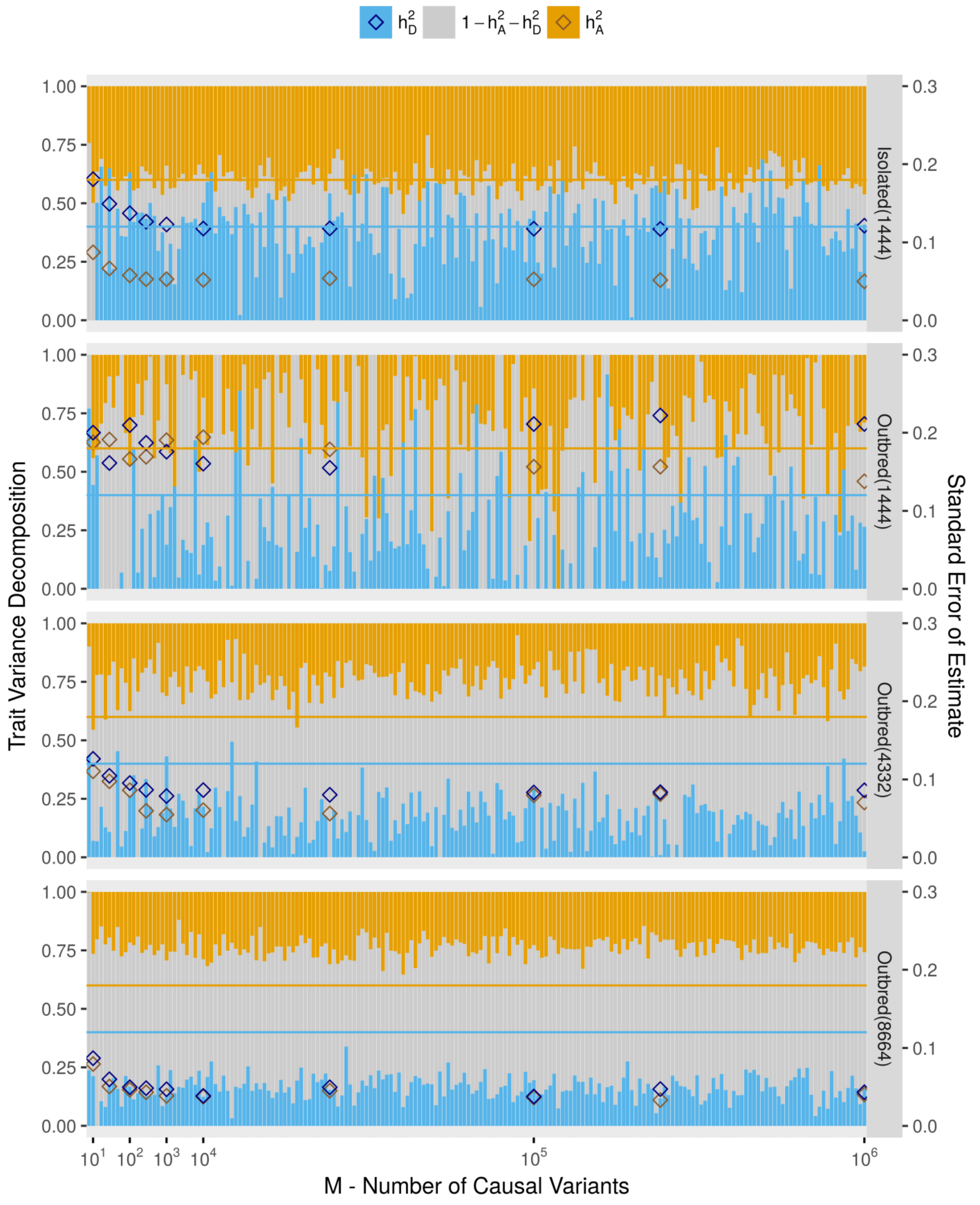

Supplementary Figure 2.
Identic to Supplementary Figure 1 apart from here K and D are calculated using roughly 170,000 variants present in all villages in Cilento, causal variants are selected to have MAF > 0.01 (Causal Variant Scenario B).

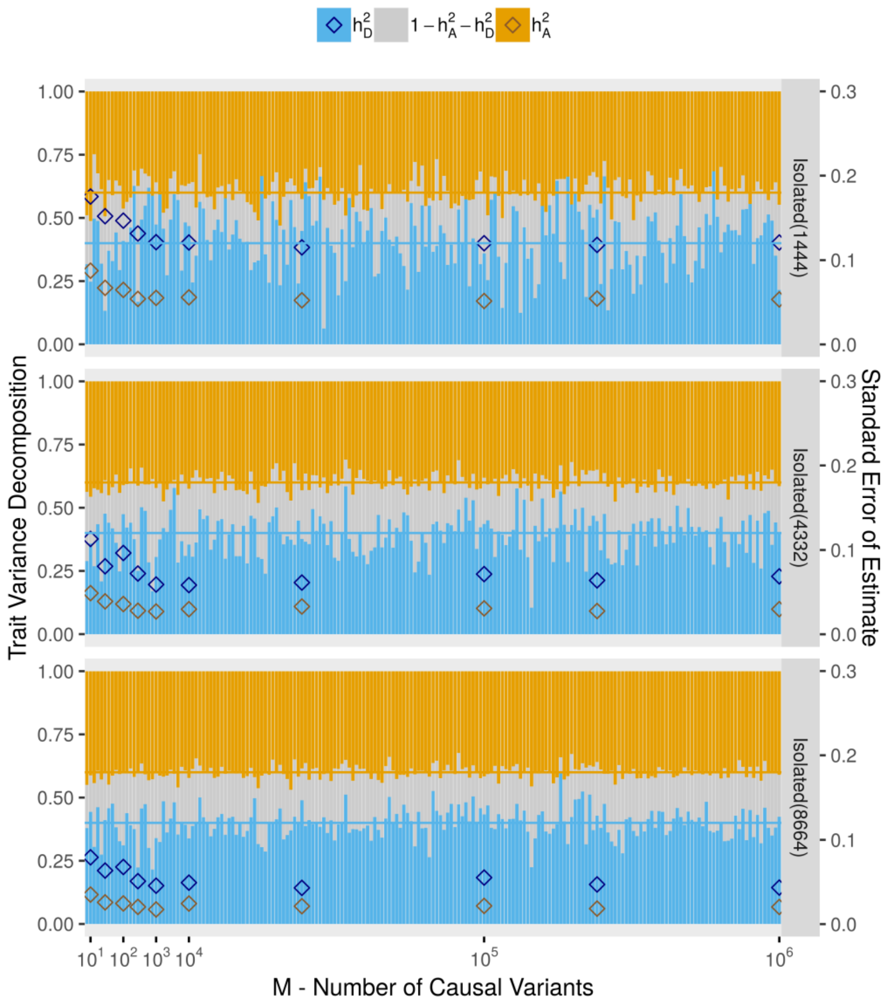

Supplementary Figure 3.
Comparison of heritability estimates from a single simulated isolated population with populations constructed by combining isolated populations.
Identic to Figure 3 in the main text but here causal variants are selected to have MAF > 0.01 (Causal Variant Scenario B).

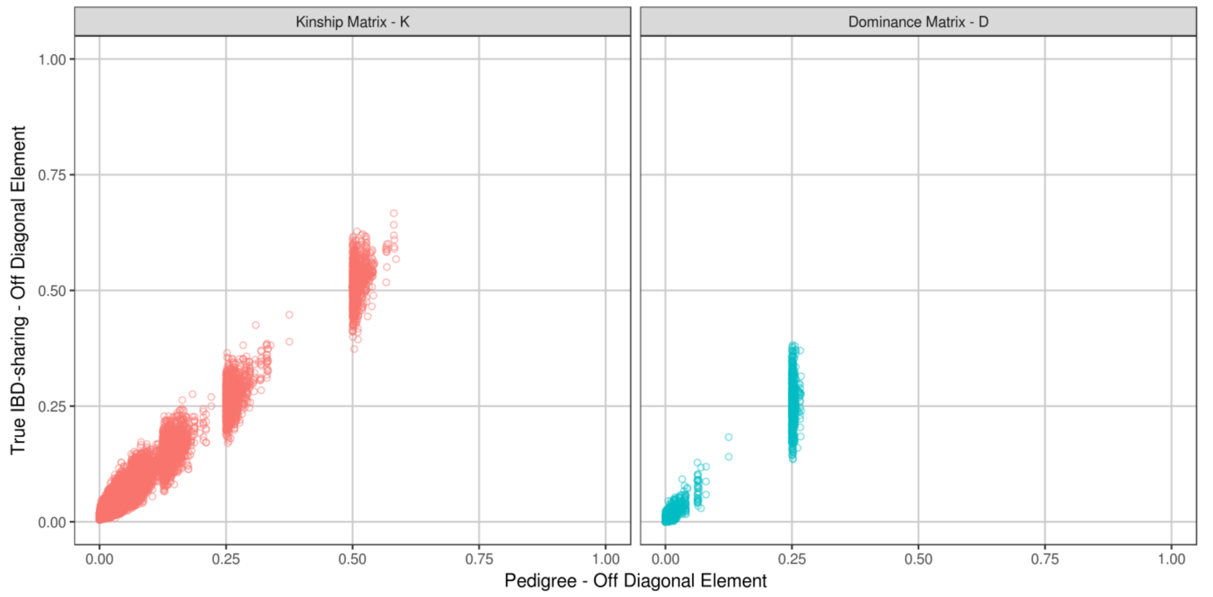

Supplementary Figure 4a.
Comparison of off-diagonal elements of the matrices K and D calculated on the simulated isolated population ‘Isolated(1444)’. K and D are calculates either from the true proportions of IBD-sharing that occurred during the simulation of the data or from the pedigree information of Cilento.

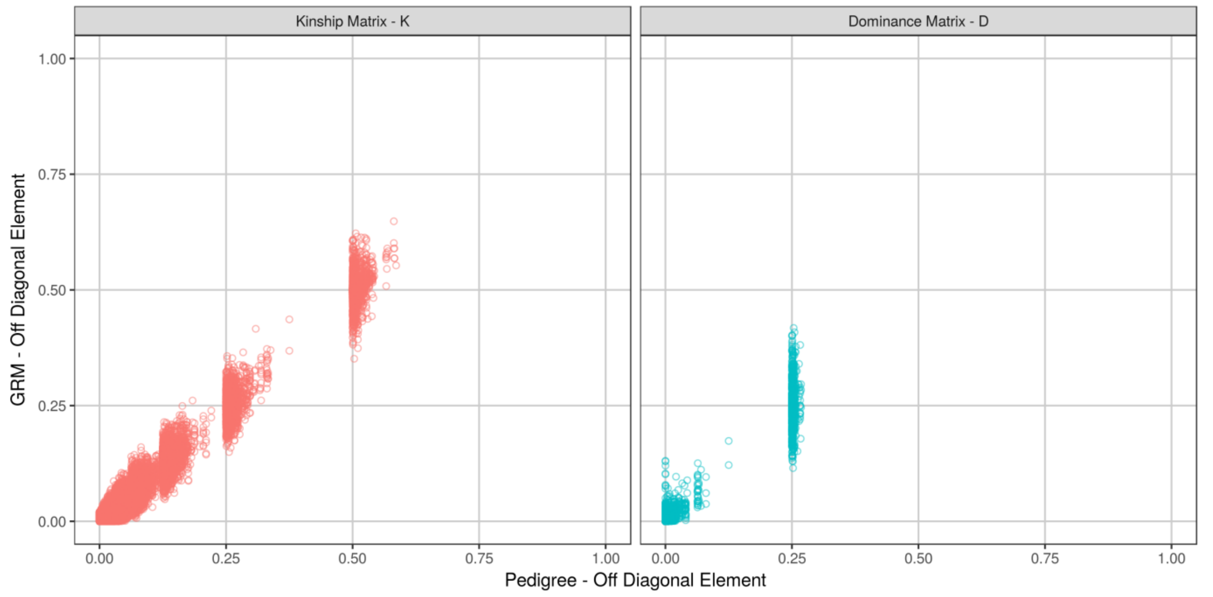

Supplementary Figure 4b.
Comparison of off-diagonal elements of the matrices K and D calculated on the simulated isolated population ‘Isolated(1444)’. K and D are calculates either as genetic relationship matrices (GRMs) or from the pedigree information of Cilento.

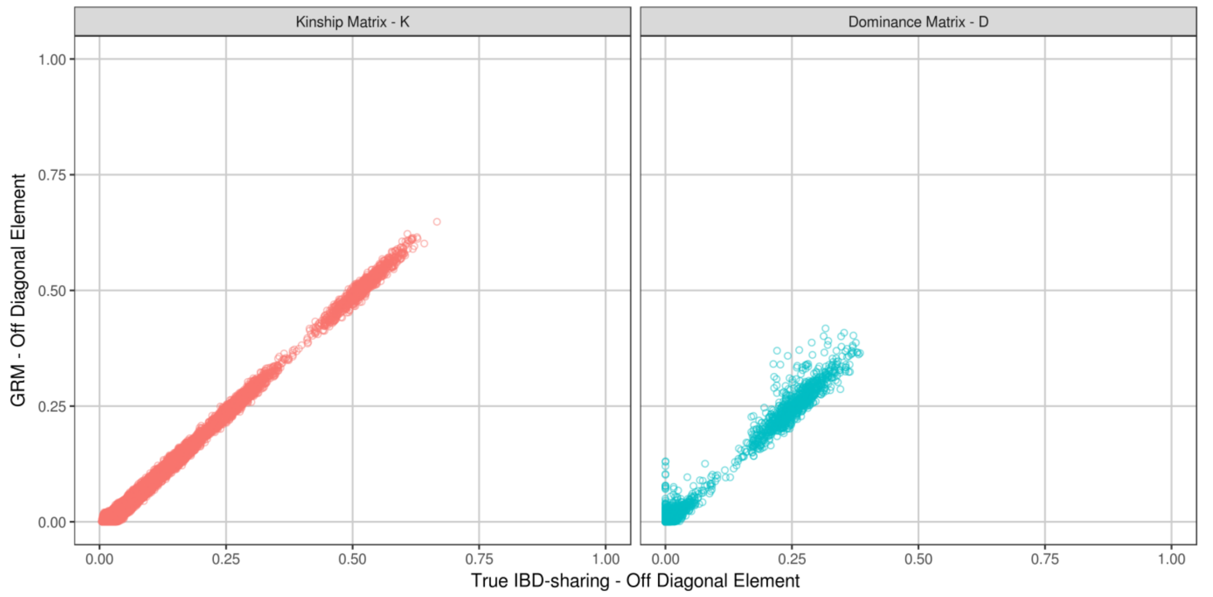

Supplementary Figure 4c.
Comparison of off-diagonal elements of the matrices K and D calculated on the simulated isolated population ‘Isolated(1444)’. K and D are estimated either as genetic relationship matrices (GRMs) or from the true proportions of IBD-sharing that occurred during the simulation of the data.

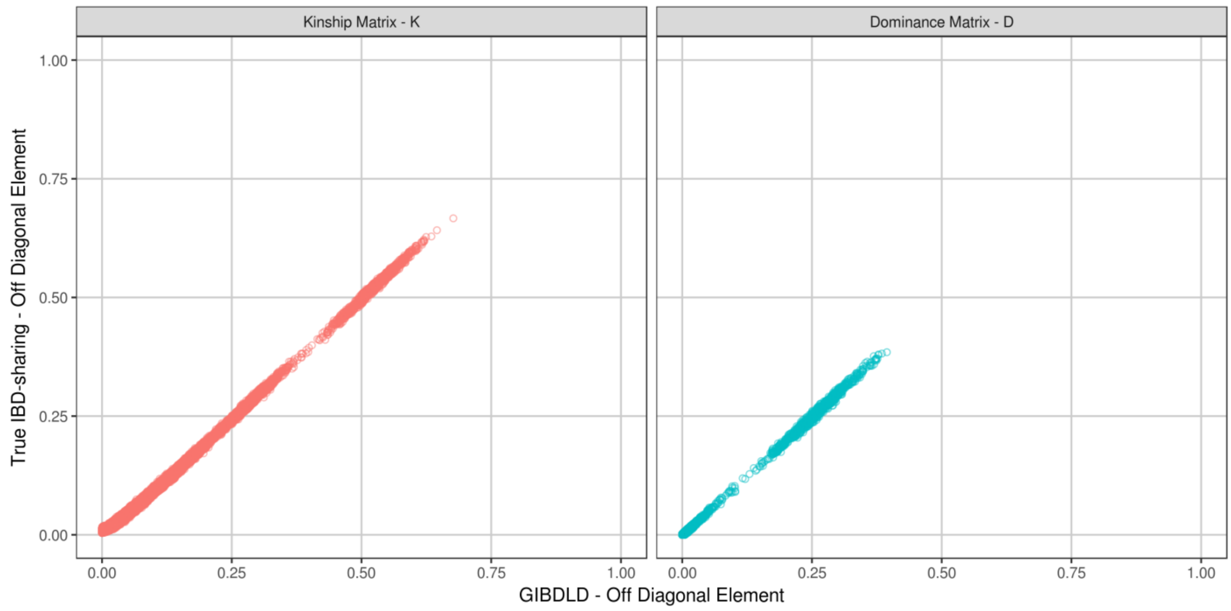

Supplementary Figure 4d.
Comparison of off-diagonal elements of the matrices K and D calculated on the simulated isolated population ‘Isolated(1444)’. K and D are estimated using either the software GIBDLD or the true proportions of IBD-sharing that occurred during the simulation of the data. Off-diagonal elements estimated by IBDLD were equally similar.

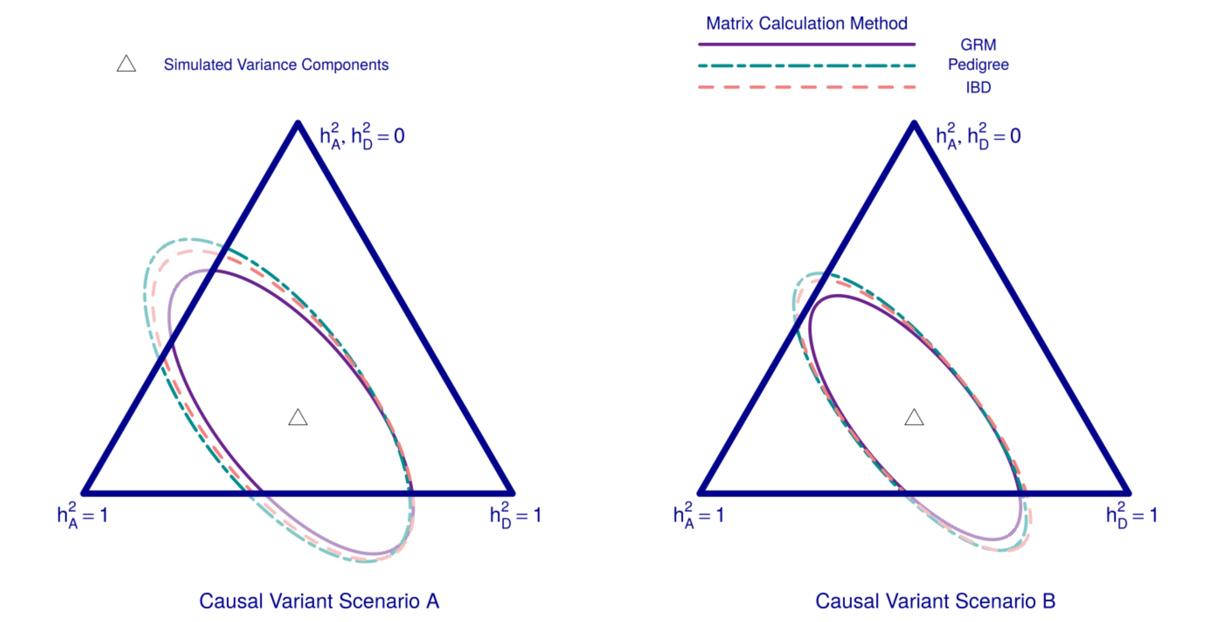

Supplementary Figure 5.
Comparison of strategy for computing matrices K and D for the Isolated(1444) population.
Identic to Figure 4 in the main text apart from here, phenotypes are simulated from a small set of causal variants (M=100).

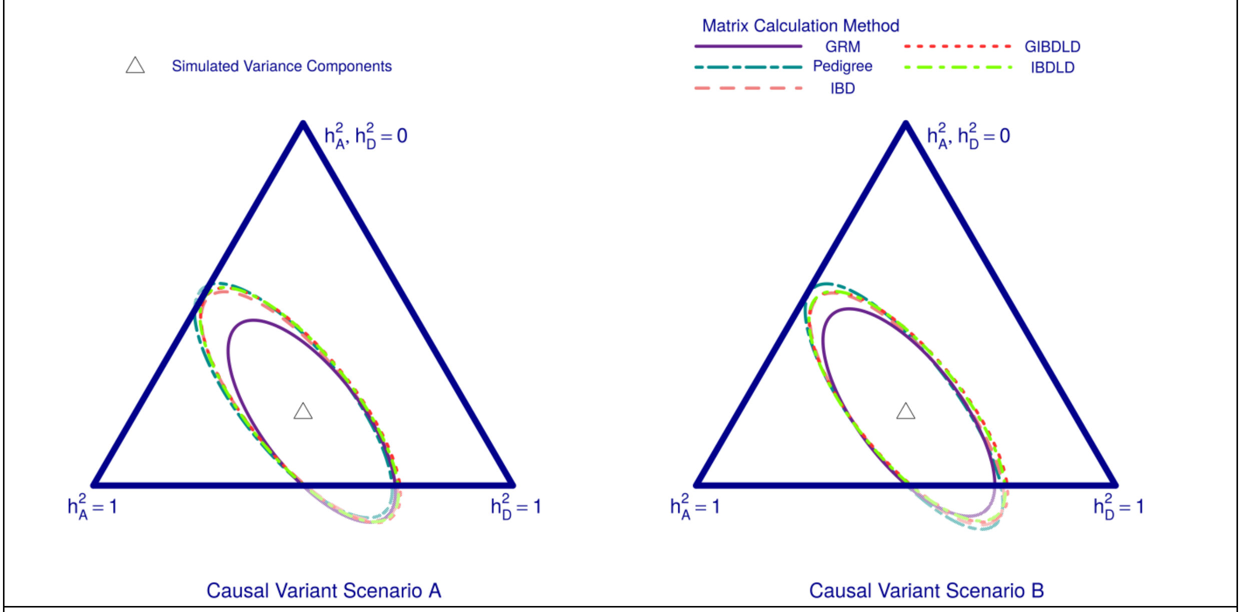

Supplementary Figure 6a.
Comparison of strategy for computing matrices K and D for the Isolated(1444) population.
The set of roughly 170,000 SNPs present in the Cilento isolates are used here to calculate K and D for the Isolated(1444) population, either as GRMs or using the IBDLD or GIBDLD software. Estimates using either pedigree information or recorded IBD-sharing from the simulating are also given.
Here, phenotypes are simulated from a large set of causal variants (M=100,000).

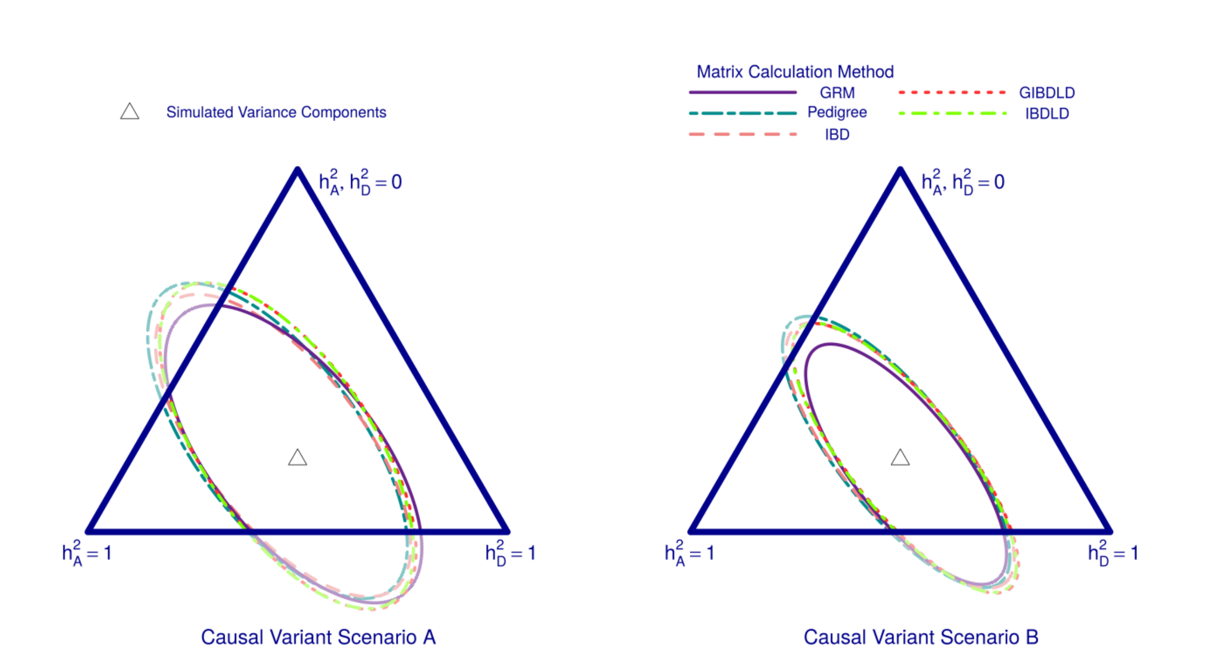

Supplementary Figure 6b.
As Supplementary Figure 6a, but here phenotypes are simulated from a small set of causal variants (M=100).

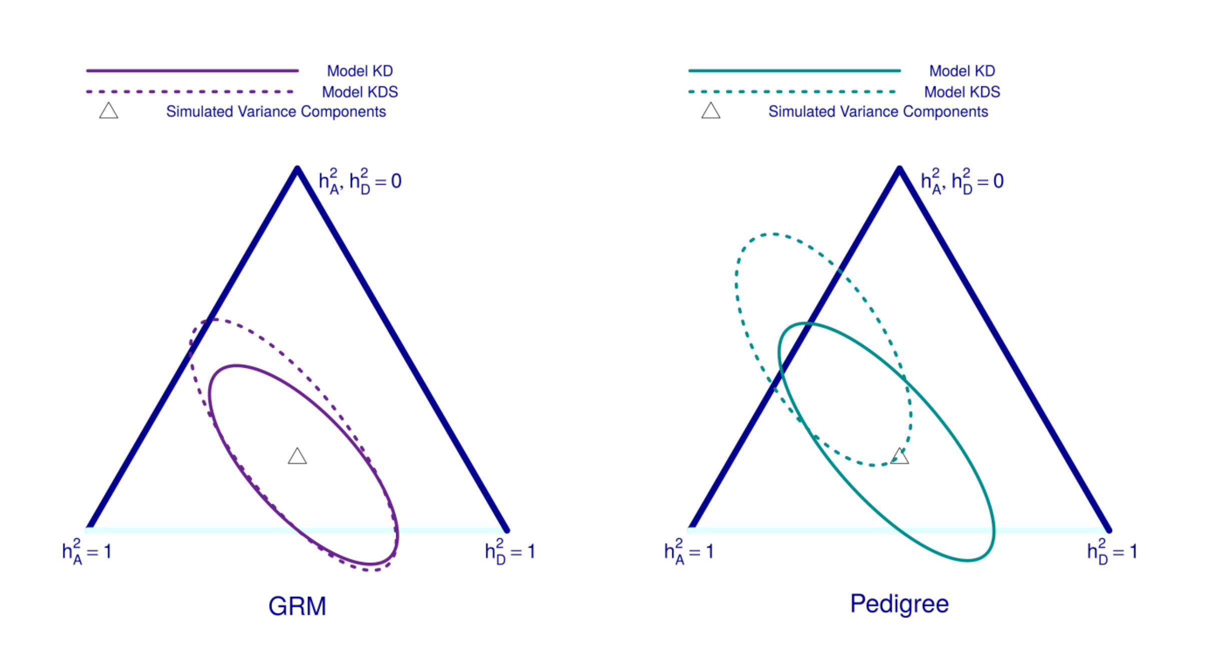

Supplementary Figure 7a.
Identic to Figure 5 in the main text but with simulated setting : $h_A^2 = 0.4,\ h_D^2 = 0.4,\ h_S^2 = 0.0$
Comparison of estimates of h_A^2 and h_D^2 under models with and without a shared environment component (model KDS and model KD, respectively). Matrices K and D are calculated either using GRMs or pedigree information.
Here, phenotypes are simulated from a large set of causal variants ($M = 100{,}000$).

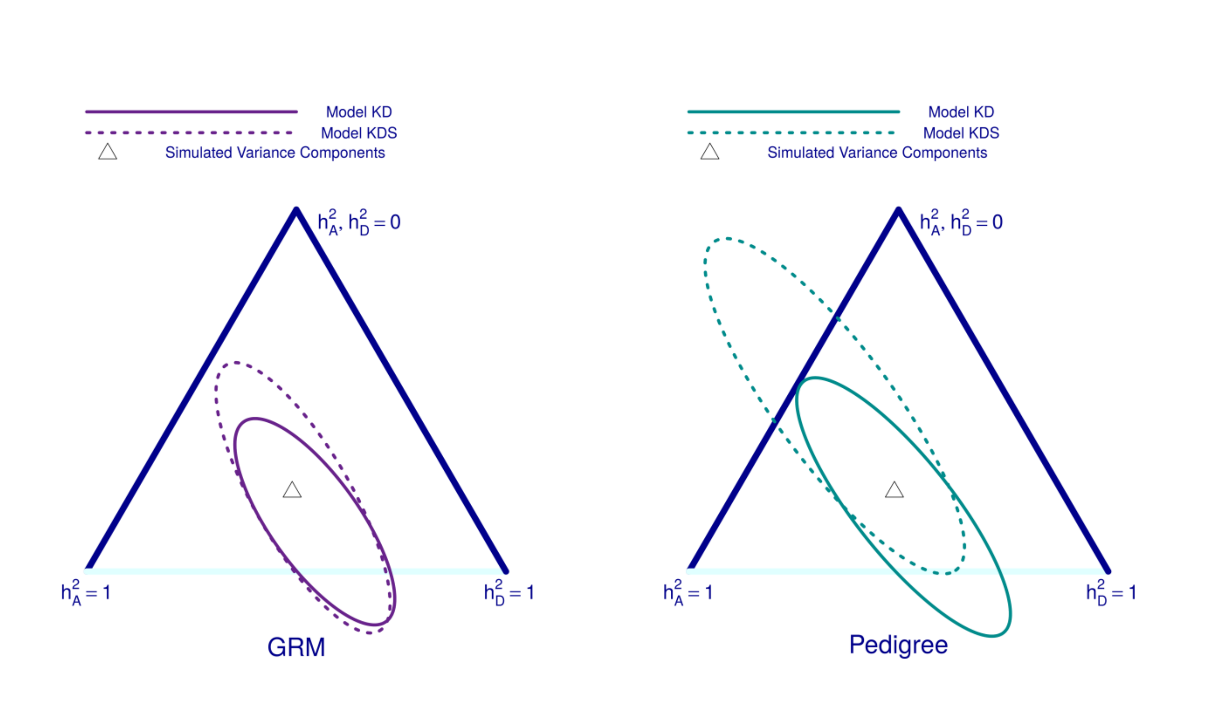

Supplementary Figure 7b.
Simulated setting : $h_A^2 = 0.4,\ h_D^2 = 0.38,\ h_S^2 = 0.02$

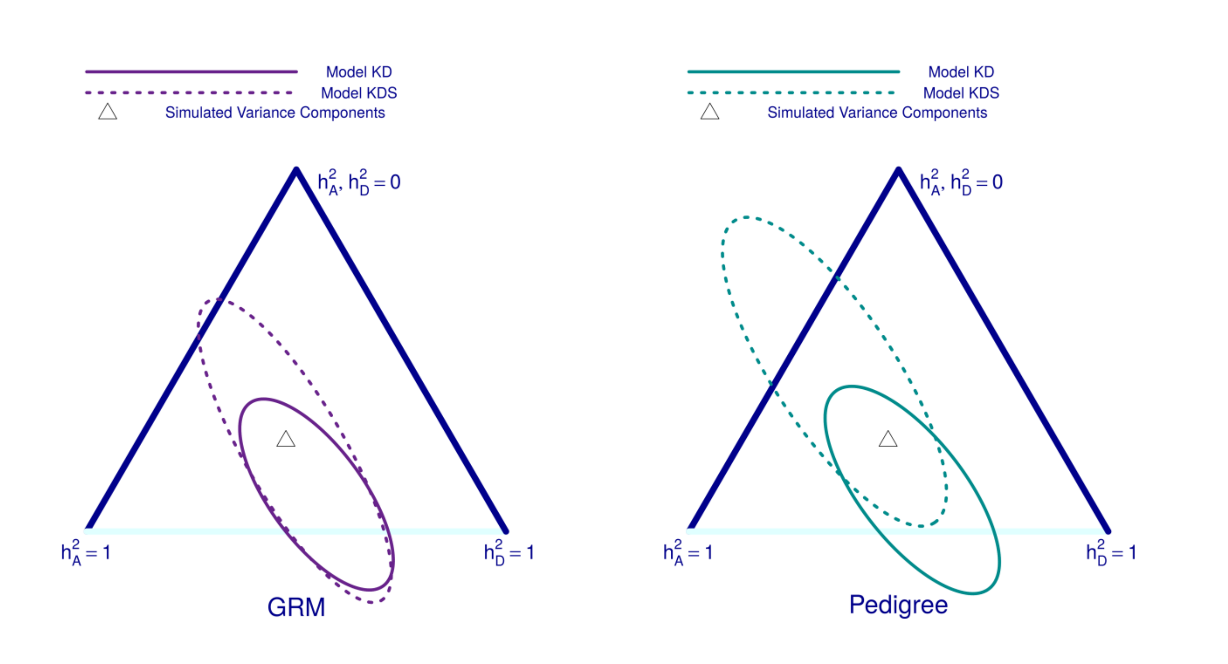

Supplementary Figure 7c.
Simulated setting : $h_A^2 = 0.4,\ h_D^2 = 0.35,\ h_S^2 = 0.05$

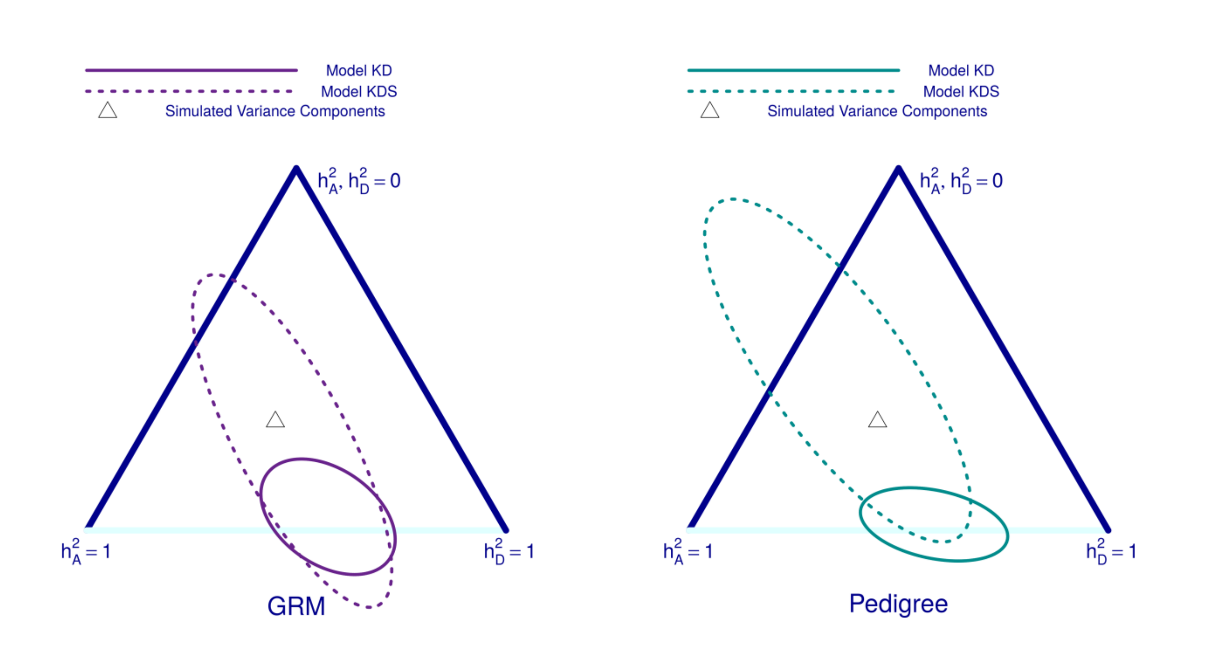

Supplementary Figure 7d.
Simulated setting : $h_A^2 = 0.4,\ h_D^2 = 0.3,\ h_S^2 = 0.1$

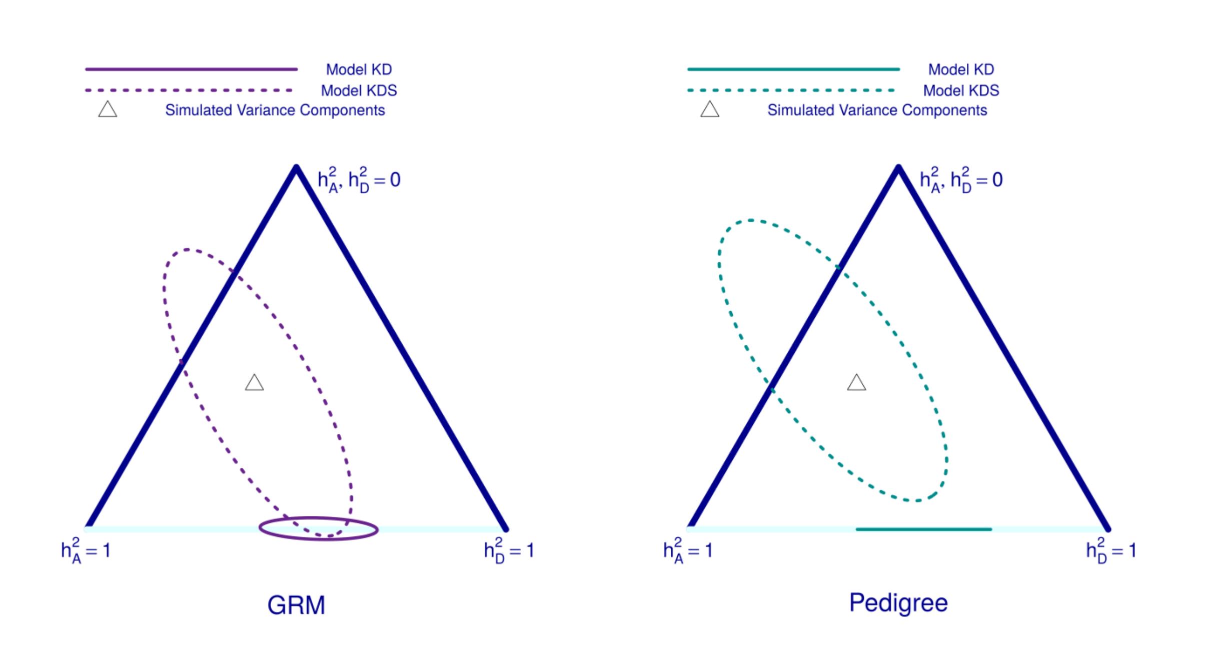

Supplementary Figure 7e.
Simulated setting : $h_A^2 = 0.4,\ h_D^2 = 0.2,\ h_S^2 = 0.2$
Completely the same as Figure 5 in the main text but is given here also for continuity with other Supplementary Figures.

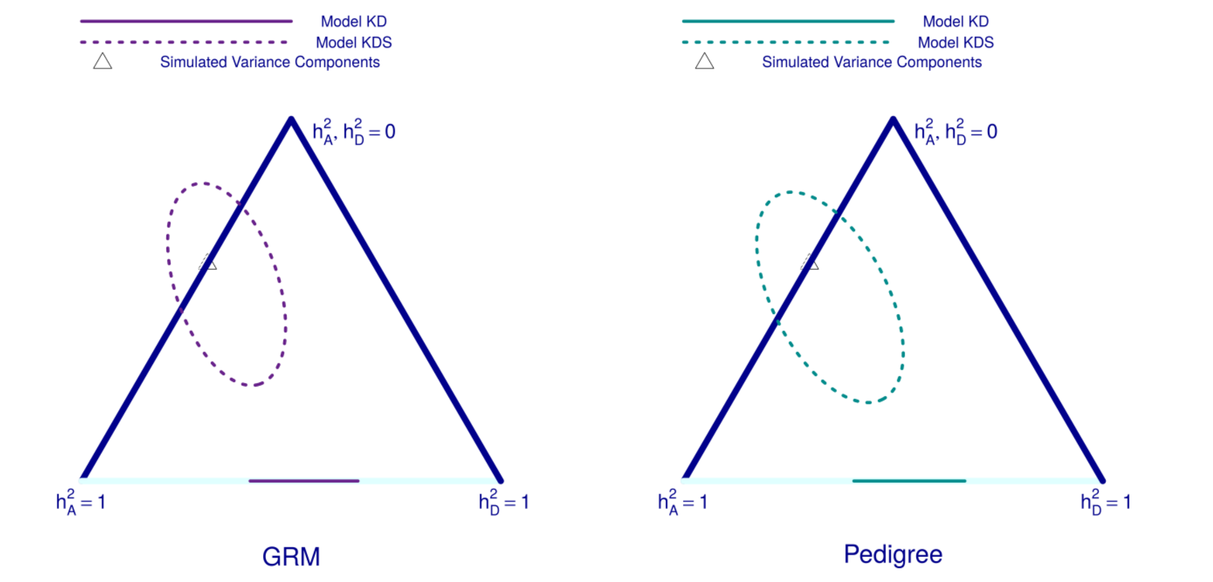

Supplementary Figure 7f.
Simulated setting : $h_A^2 = 0.4,\ h_D^2 = 0.0,\ h_S^2 = 0.4$

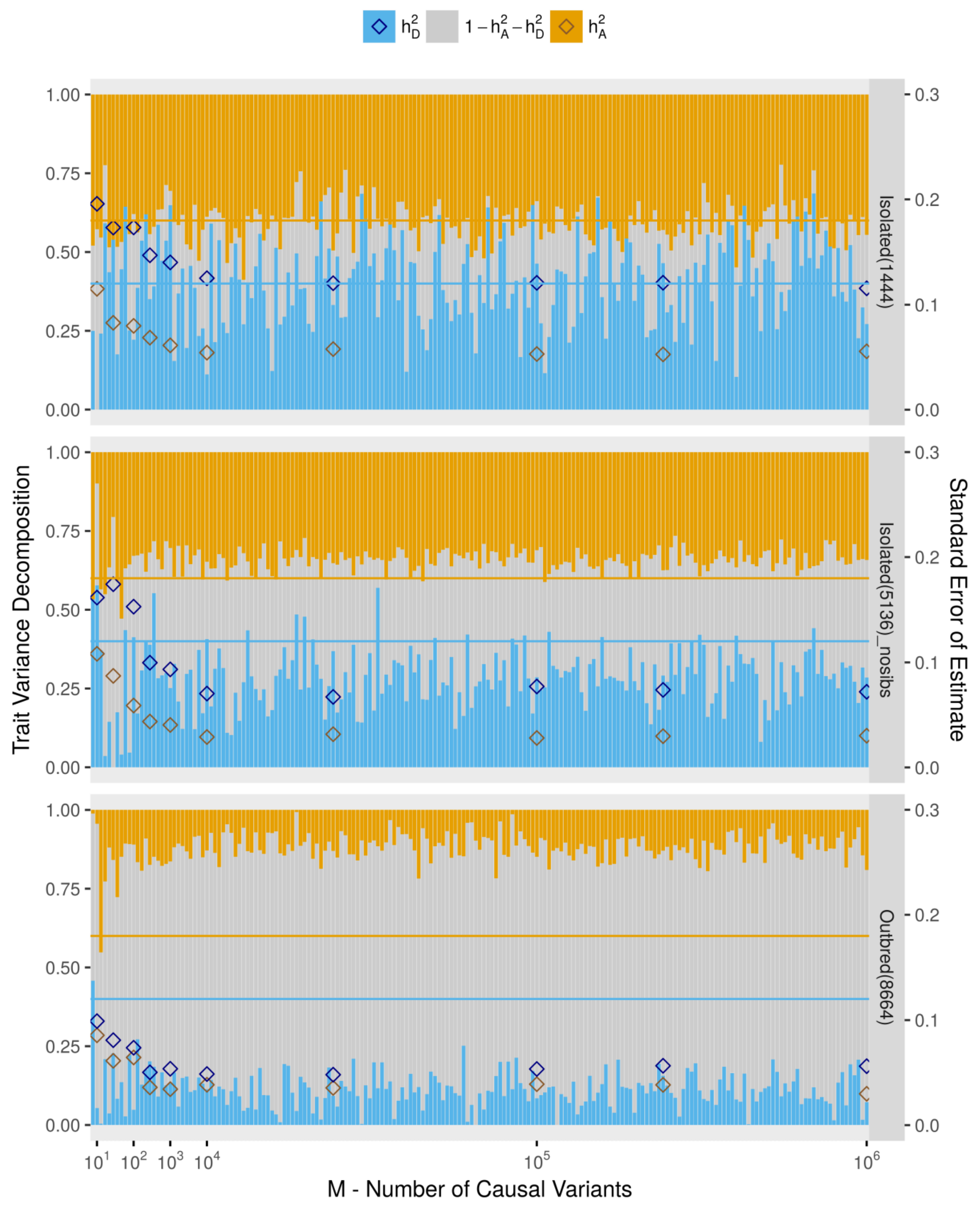

Supplementary Figure 8a.
Comparison of heritability analysis for three simulated populations.
Phenotypes are simulated under the setting : $h_A^2 = 0.4,\ h_D^2 = 0.4$
Similar to earlier Figures but here we include the larger simulated isolated population, a composite of isolates with no sibling pairs. Causal variants are selected completely at random (Causal Variant Scenario A).

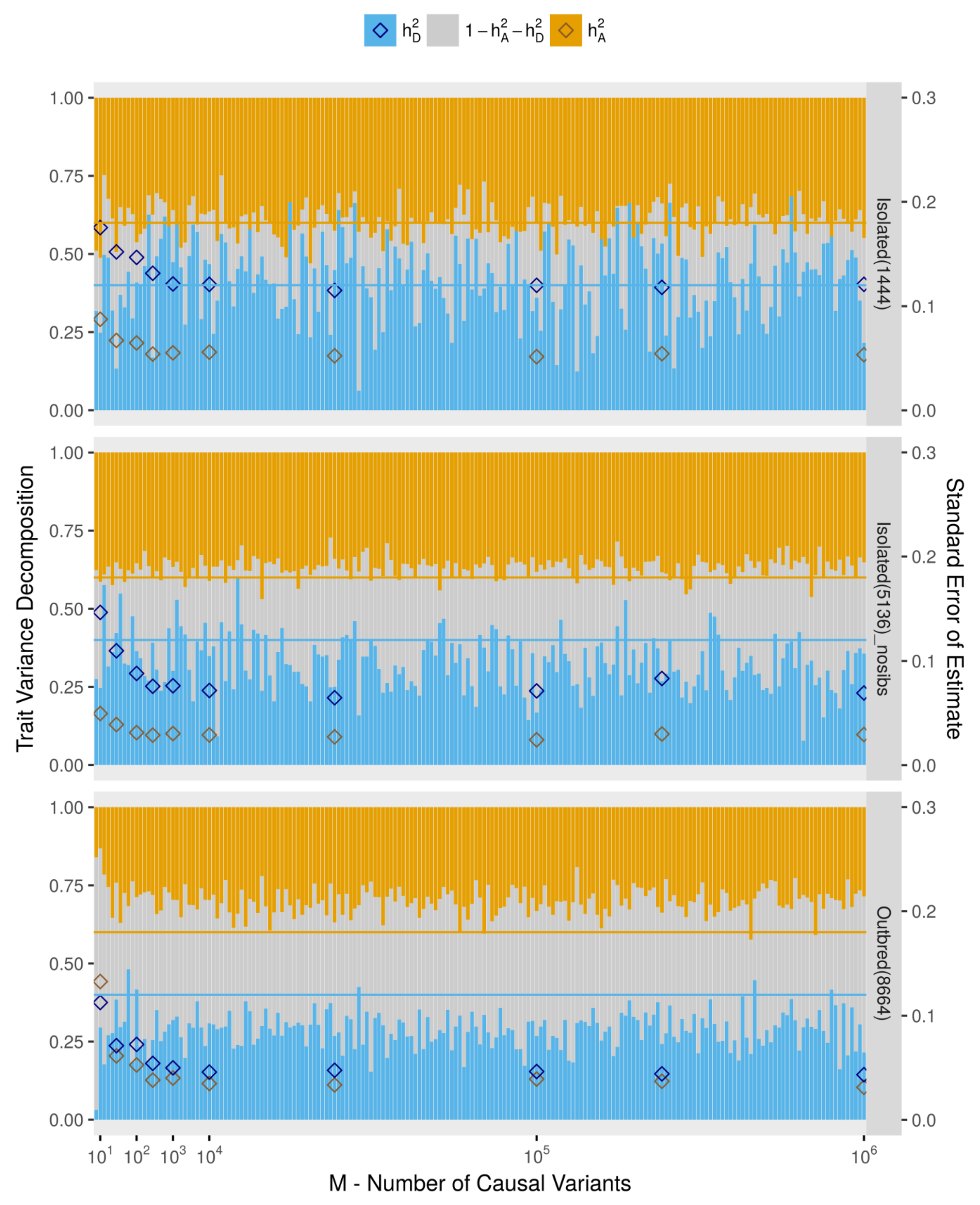

Supplementary Figure 8b.
Identic to Supplementary Figure 8a apart from here, causal variants are selected to have MAF > 0.01 (Causal Variant Scenario B).

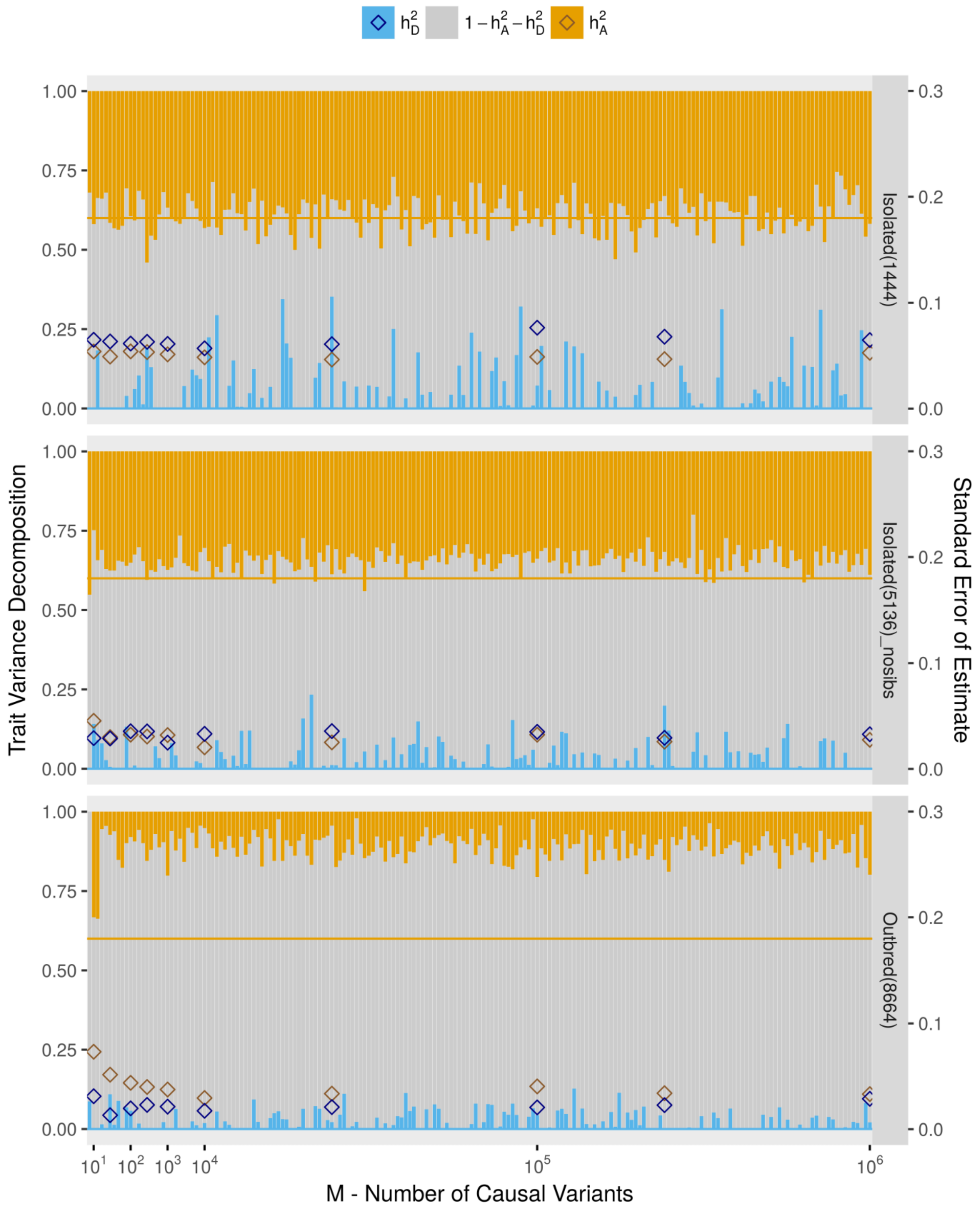

Supplementary Figure 8c.
Comparison of heritability analysis for three simulated populations.
Phenotypes are simulated under the setting : $h_A^2 = 0.4,\ h_D^2 = 0.0$
Similar to earlier Figures but here we include the larger simulated isolated population, a composite of isolates with no sibling pairs. Causal variants are selected completely at random (Causal Variant Scenario A).

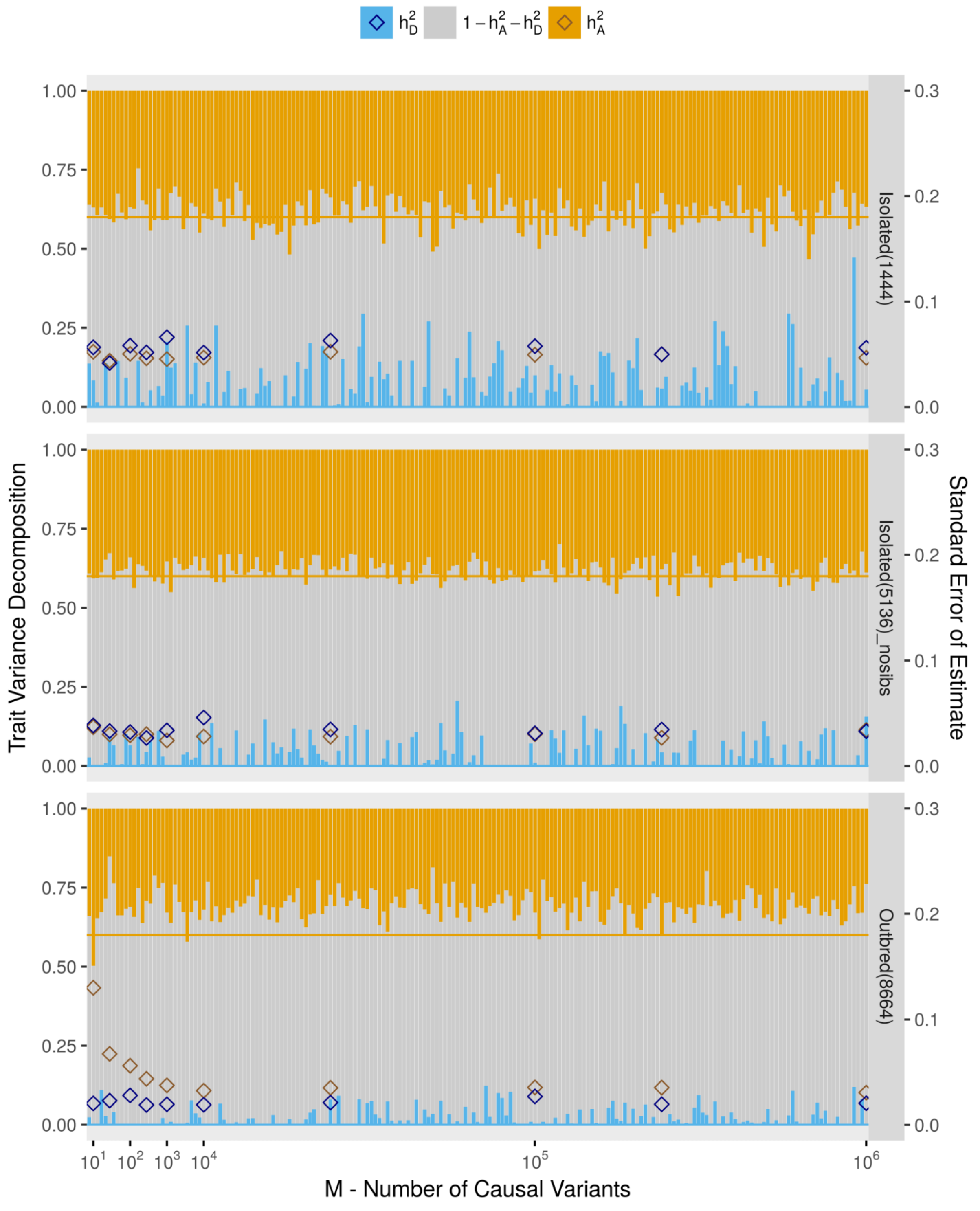

Supplementary Figure 8d.
Identic to Supplementary Figure 8c apart from here, causal variants are selected to have MAF > 0.01 (Causal Variant Scenario B).

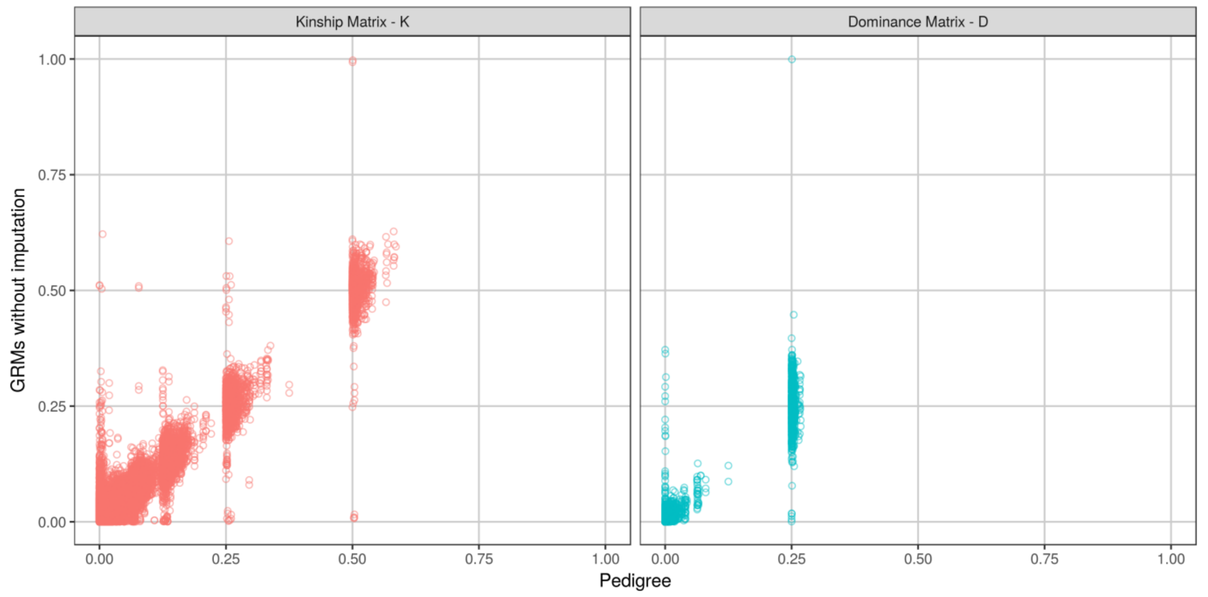

Supplementary Figure 9.
Comparison of off diagonal elements of matrices K and D calculated for the Cilento dataset using either pedigree information or genetic relationship matrices (GRMs) from the observed genotypes in Cilento.

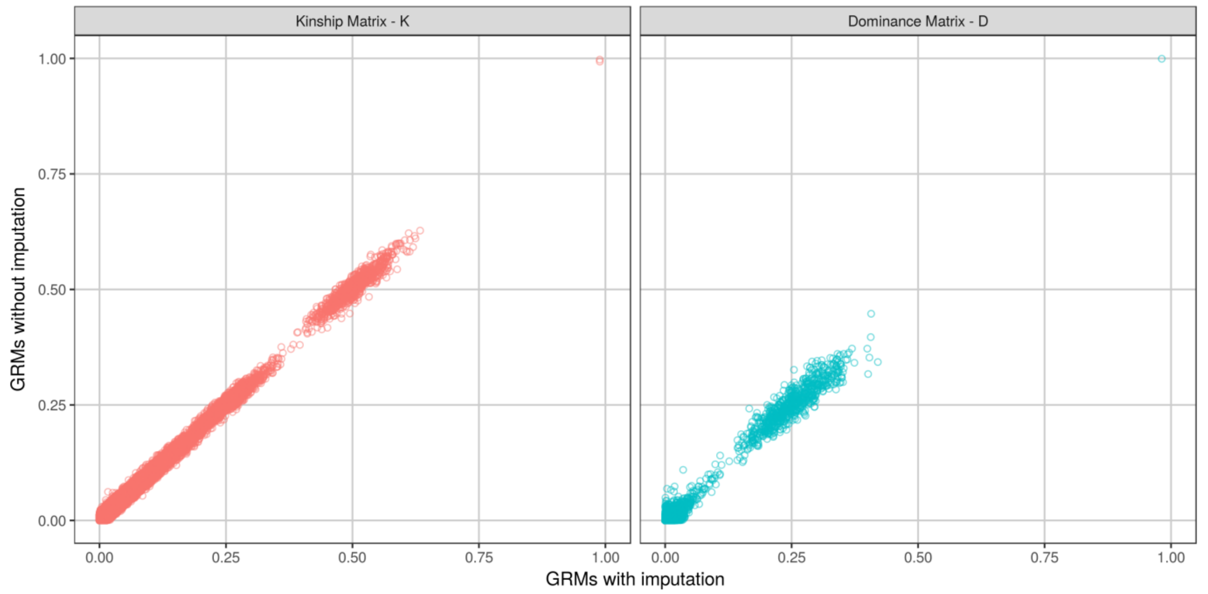

Supplementary Figure 10.
Comparison of off diagonal elements of matrices K and D calculated for the Cilento dataset using genetic relationship matrices (GRMs) before and after the inclusion of imputed variants in the Cilento dataset.

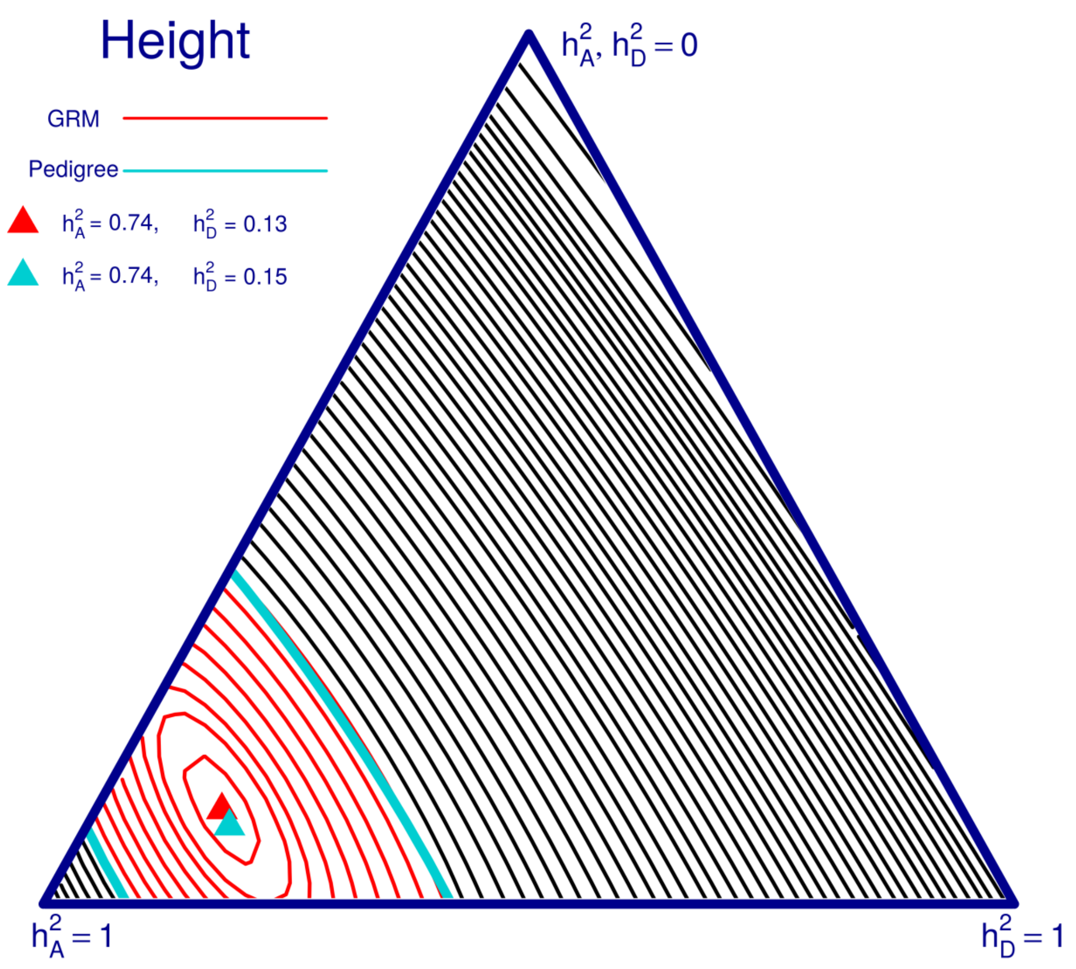

Supplementary Figure 11a.
Heritability analysis for Height in Cilento. Black contours represent the likelihood profile from the model KD (see Figure 5 in main text), with matrices K and D calculated as genetic relationship matrices (GRMs). The red zone represents the 95% confidence interval for the red maximum likelihood estimate (MLE) (red triangular peak). The corresponding MLE and 95% confidence boundary for the analysis using pedigree information to estimate K and D are added to the plot in blue.

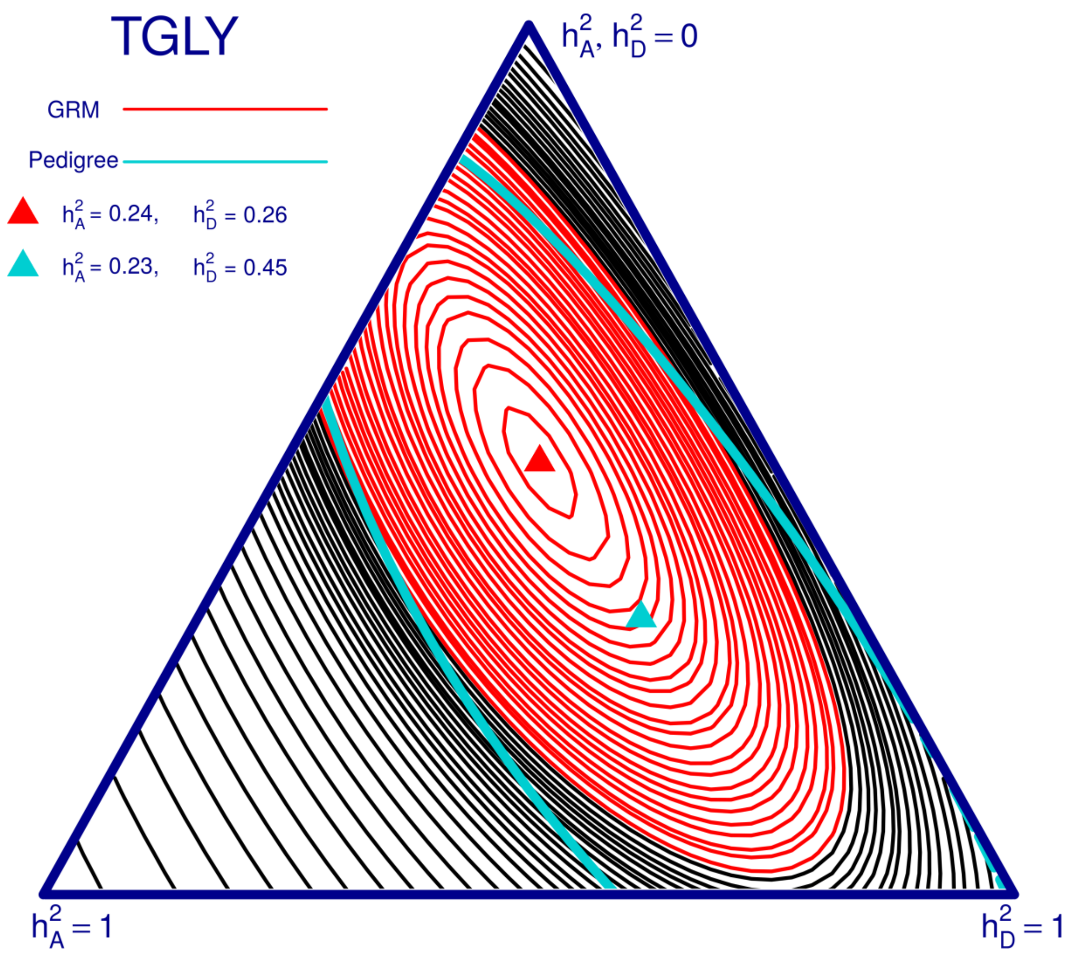

Supplementary Figure 11b.
As Supplementary Figure 11a, for the trait TGLY.

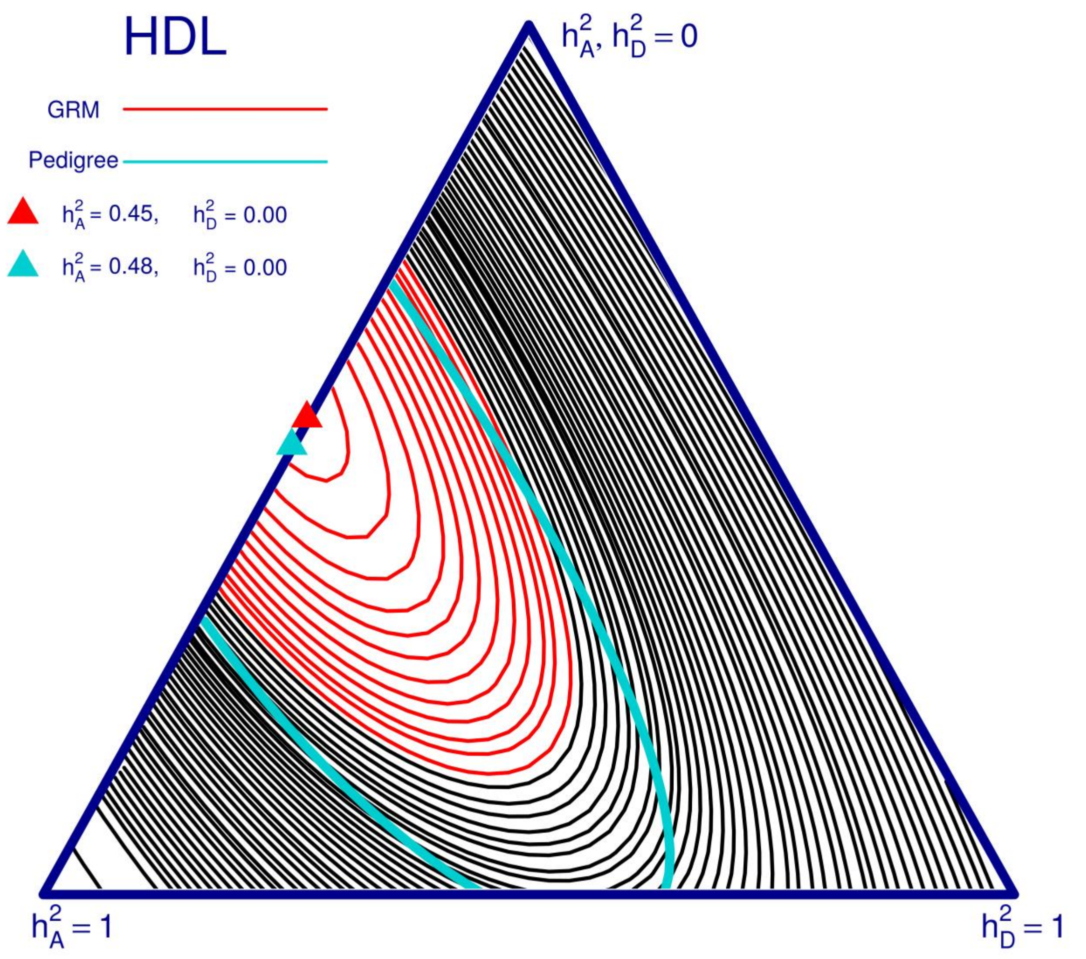

Supplementary Figure 11c.
As Supplementary Figure 11a, for the trait HDL.

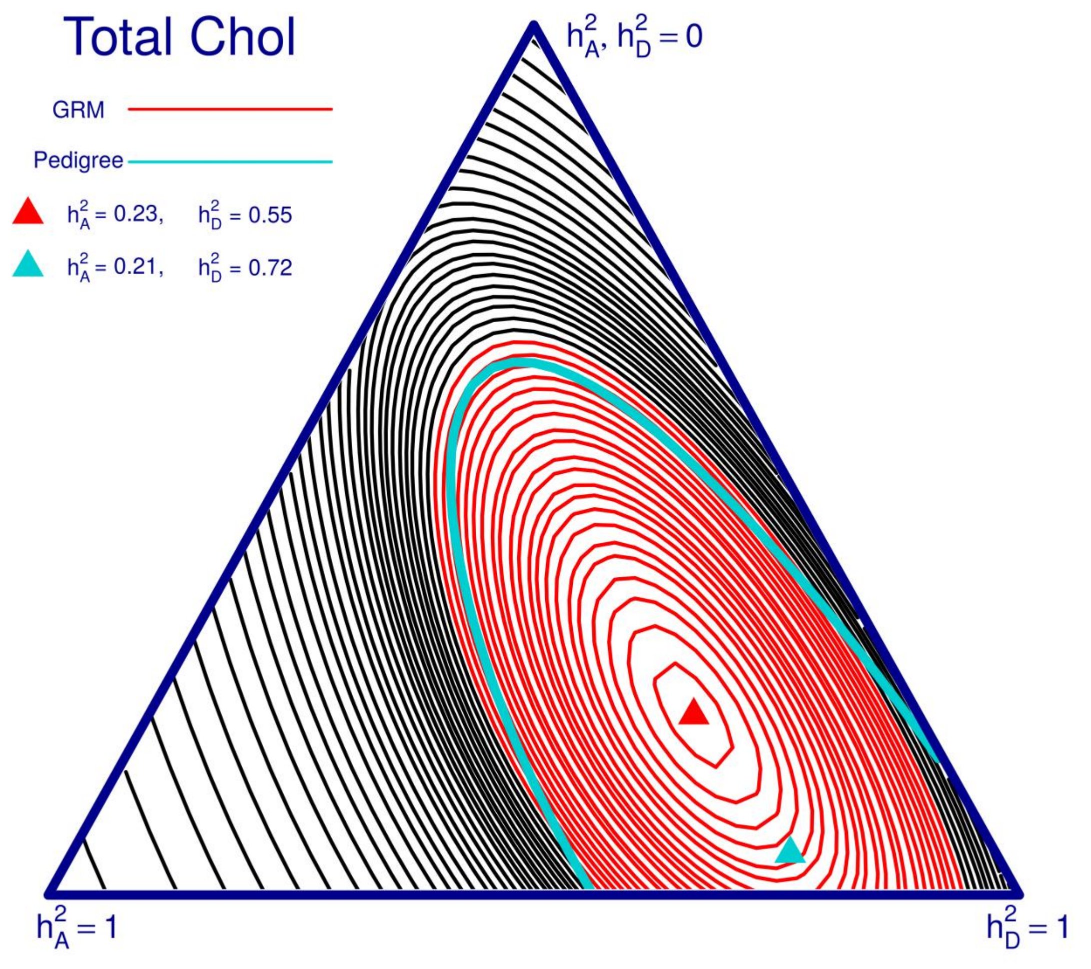

Supplementary Figure 11d.
As Supplementary Figure 11a, for the trait Total Chol.

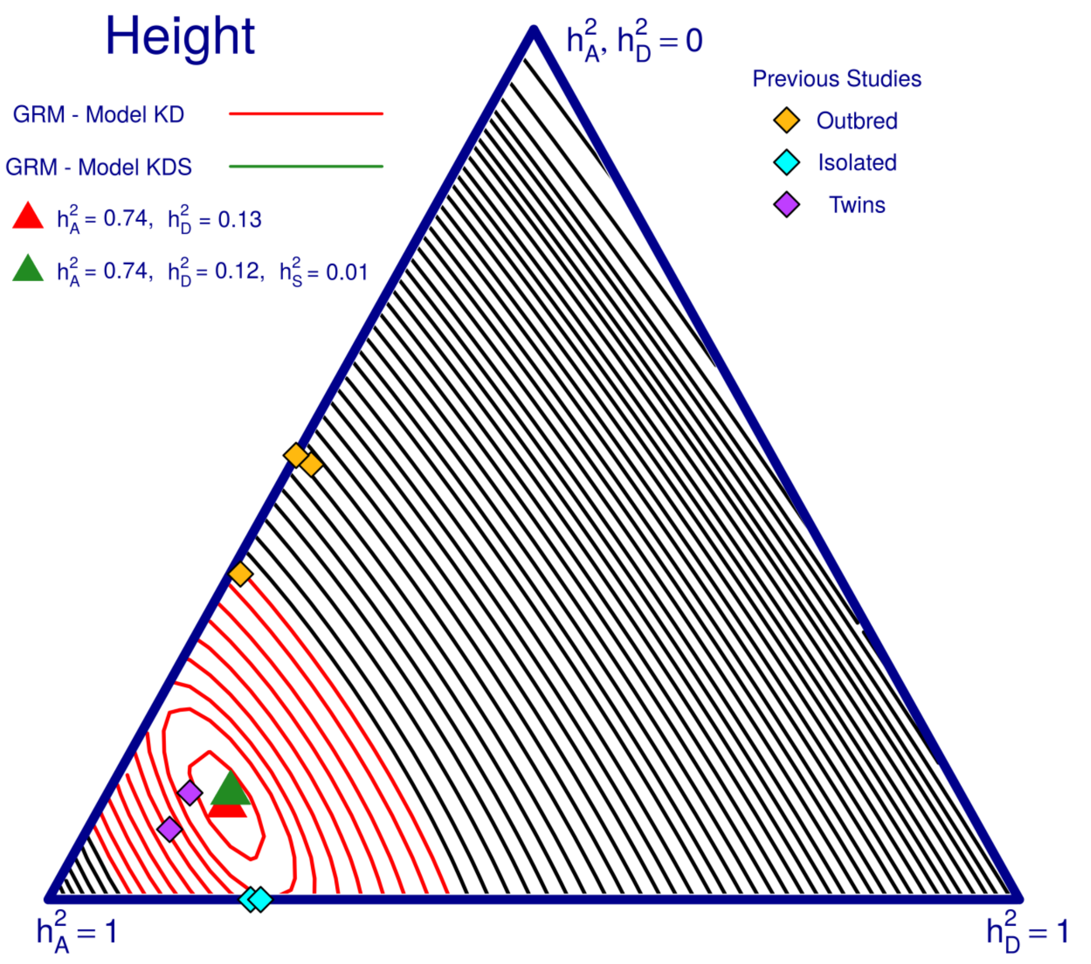

Supplementary Figure 12a.
Comparison of models KD and KDS (see Figure 7 in main text) in Cilento for the trait Height. Black contours represent the likelihood profile for the model KD, with the red zone indicating the 95% confidence interval for the red maximum likelihood estimate (MLE) (red triangular peak).
The corresponding MLE for the KDS model is added in green. We also add in the previously observed estimates from the literature (Table 1 in the main text).

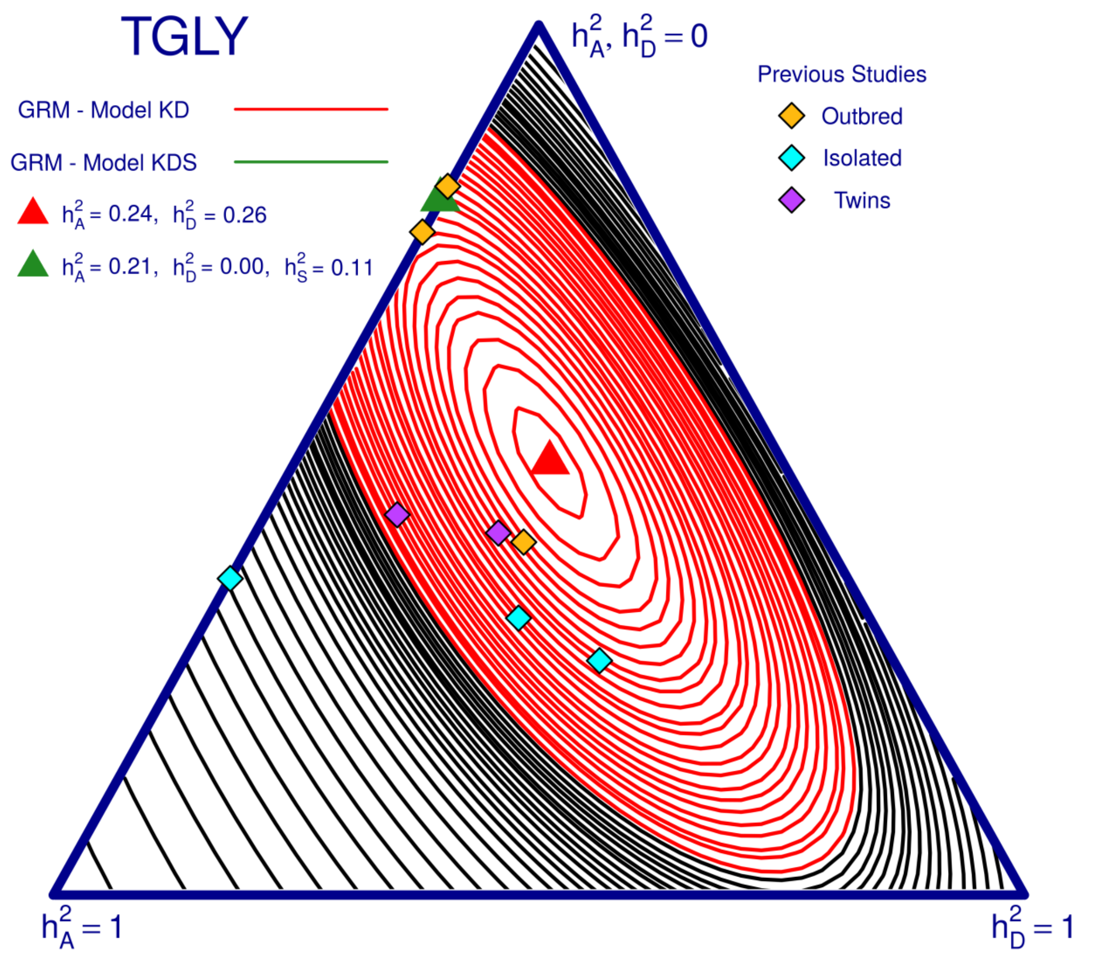

Supplementary Figure 12b.
As Supplementary Figure 12a, for the trait TGLY.

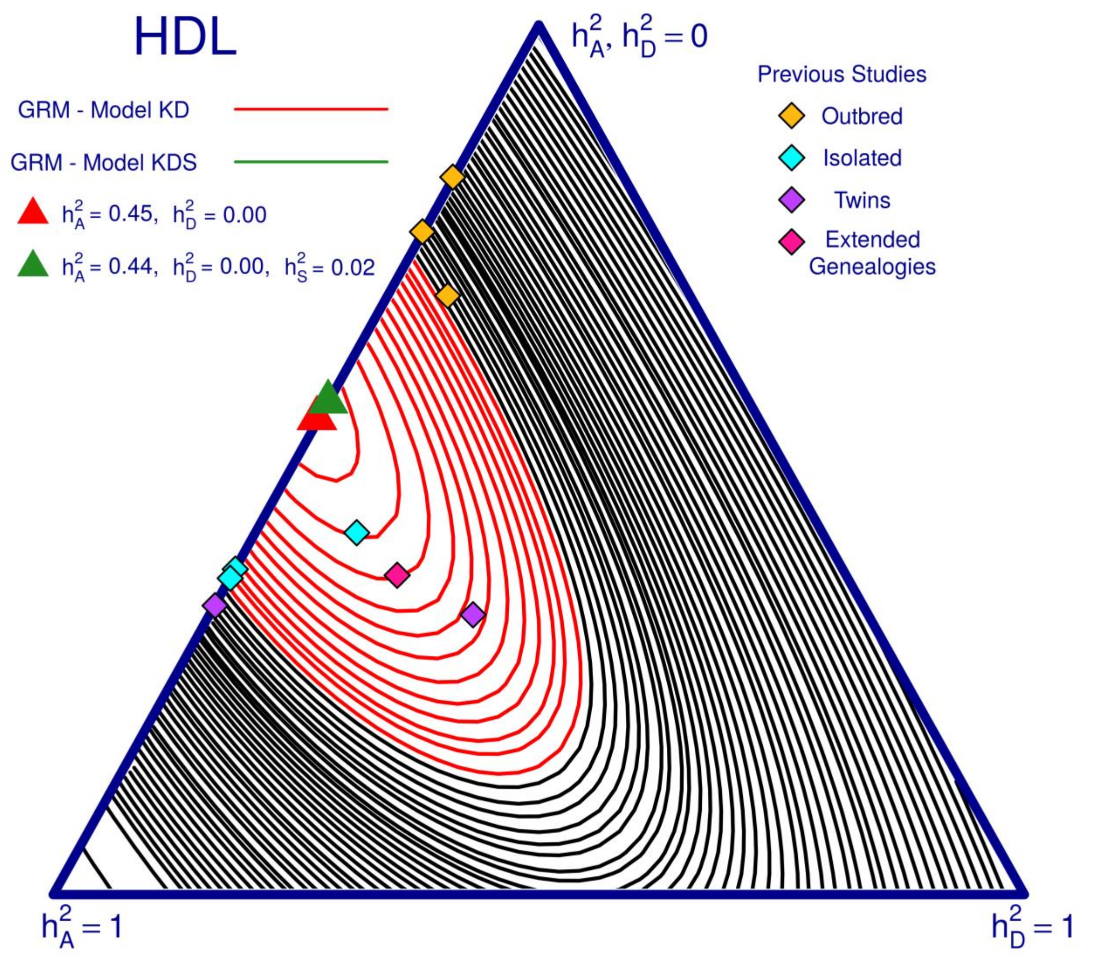

Supplementary Figure 12c.
As Supplementary Figure 12a, for the trait HDL.

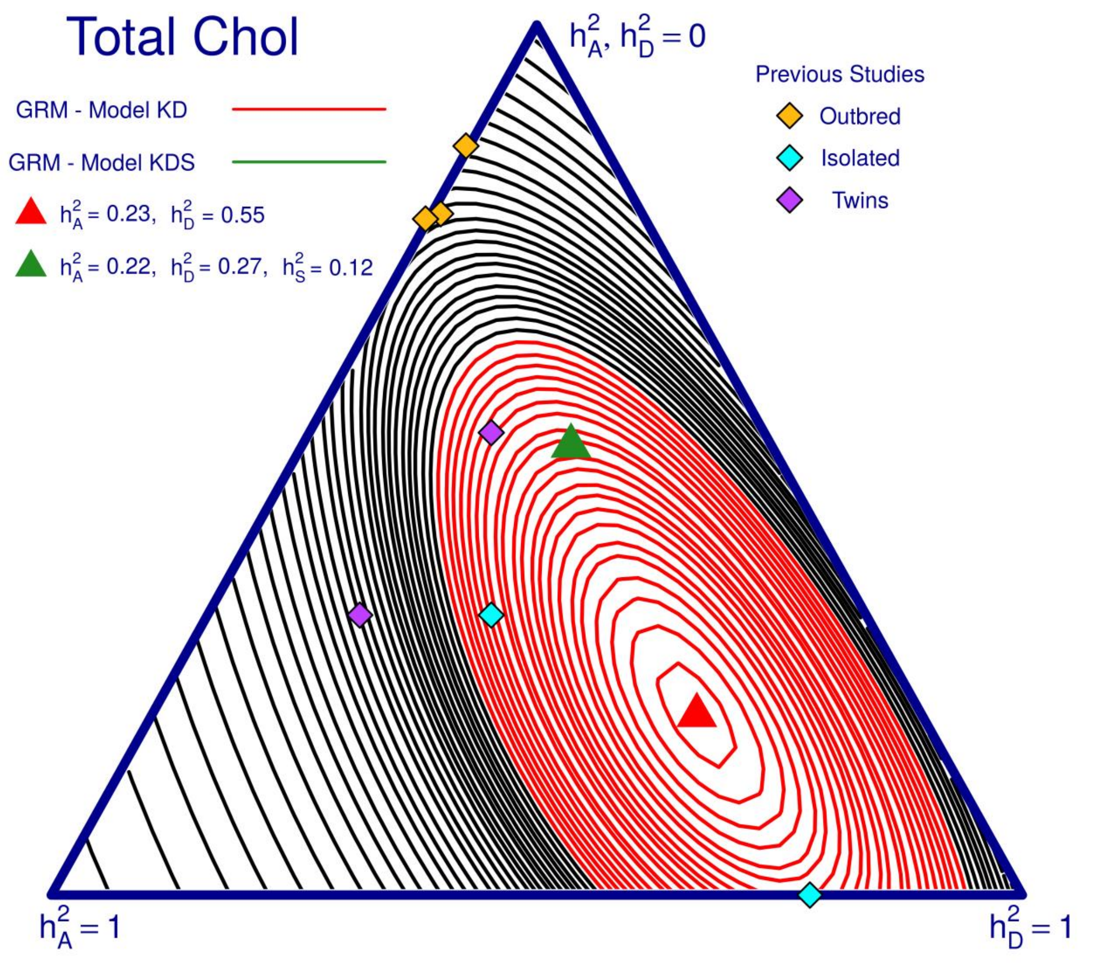

Supplementary Figure 12d.
As Supplementary Figure 12a, for the trait Total Chol.

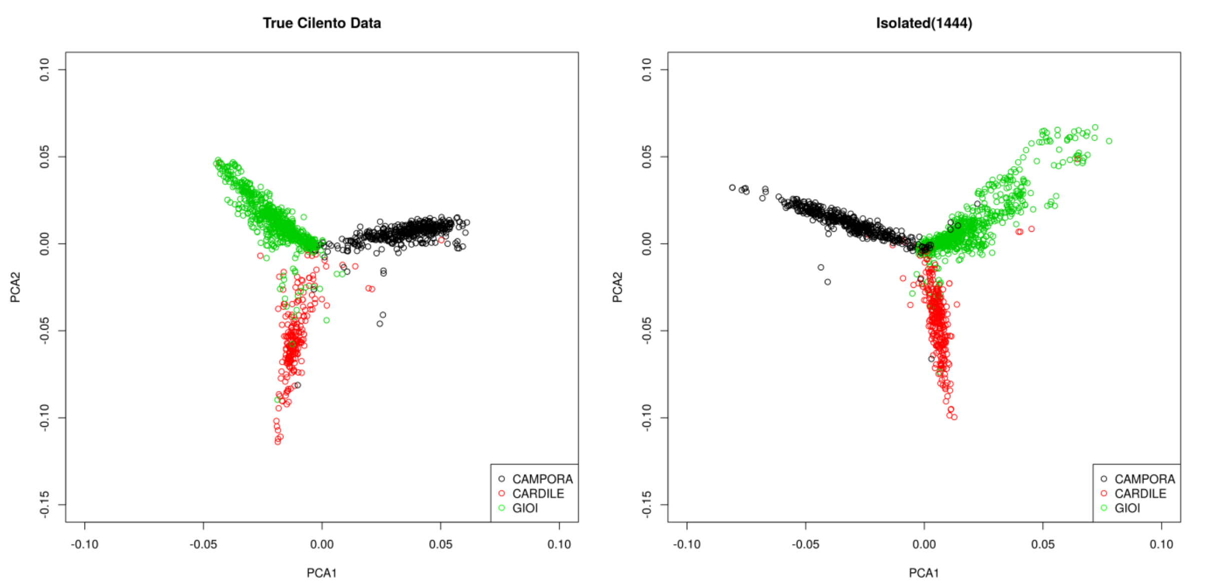

Supplementary Figure 13.
Principle components analysis, performed both in the real data of Cilento and the simulated population Isolated(1444) which aimed to mimic Cilento.

Supplementary Table 1<br>Phenotype	Mean (sd)	Non-missing values following Quality Control	Number of outliers removed	Transformation
Height	162.26(9.45)	1193	3	-
BMI	26.12(4.31)	1184	12	-
TGLY	4.77(0.52)	1326	16	Logarithmic
HDL	60.50(15.66)	1328	14	-
Total Chol	207.19(41.67)	1331	12	Adjusted for medications
LDL	118.16(35.71)	1299	12	Adjusted for medications
Abbreviations: BMI: Body-mass index; TGLY: Triglycerides; HDL: High-density lipoproteins; Total Chol: Total cholesterol; LDL: Low-density lipoproteins; sd: standard deviation.				

Summary statistics for each of the seven traits studied after the removal of outliers and application of transformations. For TGLY we performed a logarithmic transformation, and the traits Total Chol and LDL had been adjusted for a small number of individuals who were recorded as having prescriptions for lipid lowering medications.

**Supplementary Table 2**	Cilento	Isolated(1444)	Outbred(1444)	Outbred(4332)	Outbred(8664)
$var(\lambda_a)$	1.192	1.127	0.030	0.089	0.176
$var(\lambda_d)$	0.109	0.096	0.015	0.044	0.088

Comparisons of estimated variances of the eigenvalues of the matrix K (λ_a) and of the matrix D (λ_d) across different simulated populations as well as the observed data in Cilento.

References

64. Su, Z., Marchini, J. & Donnelly, P. HAPGEN2: simulation of multiple disease SNPs. *Bioinformatics* **27**, 2304-2305 (2011).
66. Raffa, J.D. & Thompson, E.A. Power and Effective Study Size in Heritability Studies. *Stat Biosci* **8**, 264-283 (2016).
69. Delaneau, O., Zagury, J.-F. & Marchini, J. Improved whole-chromosome phasing for disease and population genetic studies. *Nat Meth* **10**, 5-6 (2013).
70. O'Connell, J. *et al.* A General Approach for Haplotype Phasing across the Full Spectrum of Relatedness. *PLoS Genetics* **10**, e1004234 (2014).
71. McCarthy, S. *et al.* A reference panel of 64,976 haplotypes for genotype imputation. *Nat Genet* **48**, 1279-1283 (2016).
72. Bycroft, C. *et al.* Genome-wide genetic data on ~500,000 UK Biobank participants. *bioRxiv* (2017).
76. Purcell, S. *et al.* PLINK: a tool set for whole-genome association and population-based linkage analyses. *Am J Hum Genet* **81**, 559-75 (2007).
